# CHIMA: Design and Evaluation of a Lightweight Cryptographic System for IoT Applications

**DOI:** 10.3390/s26206468

**Published:** 2026-10-13

**Authors:** Gilberto da Silva e Silva, Jean Schmith, Francisco Erberto de Sousa, Tobias Klein Steyer, Taciano Ares Rodolfo, Arthur dos Santos Barbosa, Rogerio Ballestrin, Vitor Camargo Nardelli, Rodrigo Leão Mianes, Victor Emmanuel de Oliveira Gomes

**Affiliations:** 1 Center for Embedded Devices and Research in Digital Agriculture (Cedra) (EMBRAPII/SENAI-RS), São Leopoldo 93025-753, Brazil; silva.gilberto@senairs.org.br (G.d.S.e.S.); taciano.rodolfo@senairs.org.br (T.A.R.); rogerio.ballestrin@senairs.org.br (R.B.);; 2 SENAI Innovation Institute for Sensing Systems (ISI-SIM), São Leopoldo 93025-753, Braziltobias.steyer@senairs.org.br (T.K.S.); arthur.barbosa@senairs.org.br (A.d.S.B.); 3 Polytechnic School, Unisinos University, São Leopoldo 93022-750, Brazil

**Keywords:** lightweight cryptography, IoT cybersecurity, chaotic key generation, feistel network

## Abstract

Resource-constrained Internet of Things (IoT) devices motivate the investigation into cryptographic designs with measurable implementation costs. This work presents CHIMA (Cryptography cHaotic lIghtweight systeM and Authentication), an experimental system combining a key generator based on chaotic states, a Feistel cipher using the AES substitution box and a keyed bit permutation, and a Lesamnta-LW-based ciphertext check. Logistic, H’enon, and Lorenz systems are examined for key generation, and cipher variants with 64-bit and 128-bit blocks are evaluated. The study reports functional tests, avalanche measurements, NIST statistical tests, and implementation measurements on Arm Cortex-M0+, Cortex-M33, and Cortex-M7 microcontrollers. Controlled WiFi/MQTT and LoRa experiments assess communication interoperability. The results show that the chaotic key generator achieved high entropy and passed all NIST statistical tests, indicating its suitability for generating pseudorandom sequences. Based on the combined encryption and decryption times, CHIMA-64 requires 57.95–64.16% less time than PRESENT, while CHIMA-128 requires 23.60–59.36% less time than AES-128 and 13.32–47, as well as 10% less time than CLEFIA across the three platforms.

## 1. Introduction

The Internet of Things (IoT) connects physical devices to the Internet, enabling communication and data exchange across a wide range of applications, such as air quality evaluation [1], smart home, smart cities, industrial sensors, RFID-based sensing systems [2], autonomous cars [3,4], smart agriculture [5], energy-harvesting systems [6], healthcare systems [7], and many more. As the Internet of Things continues to evolve, security and privacy challenges become increasingly important. IoT devices often process sensitive data, such as personal, financial, and health information. When these systems are not adequately protected, they become vulnerable to cyberattacks that can compromise the integrity of operations, user privacy, and service availability [8].

The distributed and heterogeneous architecture of these devices, combined with their processing, storage, and energy consumption limitations, makes it difficult to implement robust security mechanisms [3,4]. The diversity of communication protocols [9,10] expands the attack surface, requiring cryptographic solutions that provide adequate security with low computational, memory, and energy requirements.

A complete cryptographic system for IoT is mainly composed of an encryption/decryption scheme and an authentication mechanism. Within the encryption/decryption scheme, secret-key generation is an important concern. An interesting alternative for secret-key generators is chaotic systems [11,12,13,14,15]. Another approach in cryptographic design is the use of time-varying keys, in which cryptographic parameters change over time rather than remaining fixed throughout the encryption process [16].

Chaotic systems are mathematical models derived from dynamical systems that are used to represent and describe many phenomena [17,18]. An important property of these systems is the sensitive dependence on initial conditions, that is, a small change in the initial state can lead to significantly different trajectories over time. However, chaotic systems are deterministic. This means that for the same initial conditions, the same parameterization, and the same number of iterations the result should be the same. These characteristics of chaotic systems may lead to pseudorandom behavior, making them potentially useful for achieving the confusion and diffusion properties required in cryptographic systems [19]. The literature presents many implementations of chaotic systems for secret-key generation [13,14,15], implementations in FPGAs [20,21] and low-cost hardware [22,23]. One concern is that the sensitivity of chaotic systems may be affected by the limitations of floating-point implementations on constrained devices [24]. However, a recent study evaluated the logistic map and Lorenz chaotic systems on three different Arm-based microcontrollers and obtained identical results under several conditions, suggesting that this approach can be used on constrained devices as long as the rounding mode chosen from IEEE 754 is the same on each device, i.e., round-to-nearest [25].

Lightweight cryptography remains an active research topic, as reflected by the number of recent surveys [3,4,8,26,27,28]. Fursan et al. [29] proposed a lightweight cryptographic algorithm inspired by Feistel networks and substitution permutation architectural methods. They compared their system with widely used cryptographic systems and achieved good results. Chakrabarty et al. [30] proposed a lightweight multilevel cryptography to enhance data security with good security improvements. Mahajan and Reddy [31] proposed a lightweight cryptography algorithm to improve gene data processing operations using blockchain. Their approach reduced cryptographic overhead by 27% compared to other existing methods. El-hajj et al. [32] analyzed 122 lightweight cryptographic algorithms on Arduino and Raspberry platforms and provided a complete map of them considering power, speed and ROM measurements. Yasmin and Gupta [33] presented a scheme to encrypt files in an energy-efficient manner that is suitable for the IoT environment. They compared with famous lightweight algorithms with good results. Qaid et al. [34] developed a lightweight cryptographic algorithm based on DNA computing for IoT devices. When comparing their approach with other encryption algorithms, the experimental tests using images presented good results. Imdad et al. [35] also presented a DNA approach and the classic PRESENT algorithm leading to the DNA-PRESENT block cipher. Their approach had a cost increase with an increase in security performance. Radhakrishnan et al. [36] compared three different lightweight cryptographic algorithms, AES-128, SPECK, and ASCON, in resource-constrained IoT devices. Their evaluation showed that SPECK outperformed the other algorithms.

Authentication is commonly incorporated into cryptographic systems to provide data integrity and source authentication. Recent works have also investigated lightweight authentication mechanisms for wireless systems with constrained communication, computational, and energy resources. For example, Yue et al. [37] proposed a tree-chain authentication scheme for ADS-B systems, combining hash-based data integrity with shared signatures for source authentication while reducing communication and computational overhead. Ahmed et al. [38] proposed a combination of lightweight cryptography and authentication for the IoT. They introduced a lightweight cryptographic scheme integrated with authentication, employing the elliptic-curve Diffie–Hellman method to perform the key distribution. They achieved a 50% reduction in execution time, 19.6 to 32 % storage reduction, and 41 % energy reduction compared to other classical algorithms. Goel et al. [39] proposed an authentication-based solution for secure IoT networks. Their simulated model outperformed other cryptosystems in terms of execution speed. Garba et al. [40] proposed a blockchain-based authentication for IoT applications. Their evaluation showed reasonable overhead and indicated suitability for constrained devices. Masud et al. [41] proposed a lightweight and anonymity-preserving user-authentication scheme for resource-constrained IoT-based digital health networks. They evaluated the scheme against several classes of attacks and reported resistance to the considered attacks. It also has low computational and communication costs compared to state-of-the-art approaches at that moment. Sun et al. [42] proposed a lightweight authentication, encryption, and key distribution approach for IoT devices using neural cryptosystems and binary latent space. Their results suggest that neural approaches may be applicable to security and privacy in IoT systems.

Other works in the literature have implemented the lightweight cryptography algorithms in hardware. Nguyen et al. [43] implemented the ASCON lightweight cryptography algorithm on an FPGA and evaluated its hardware performance and resource utilization. Vijaykumar et al. [44] designed a novel lightweight mutual authentication protocol using a multifunctional digital logic-based encoder architecture for RFID-based IoT systems. They compared their results with the state-of-the-art protocols, demonstrating the application for lightweight applications.

Many of the works cited above evaluate their proposals through a combination of functional experiments, performance measurements, and comparisons with existing cryptographic or authentication schemes [26,32,37,45]. Many of these works use the NIST Statistical Test Suite (STS) to evaluate the statistical properties of generated sequences [46]. NIST SP 800-22 specifies a suite of statistical tests for evaluating the properties of binary sequences [47]. In addition to NIST tests, comparative analysis with widely adopted state-of-the-art algorithms is commonly found in the literature [29,31,32].

Statistical tests and implementation benchmarks provide complementary but limited evidence about a proposed cryptographic design. NIST SP 800-22 examines statistical properties of bit sequences; it does not certify a cipher or replace cryptanalysis [47]. Functional tests assess implementation consistency, whereas resistance to adversarial attacks requires analysis of the construction, key generation, operating mode, and surrounding protocol. A useful experimental evaluation must therefore distinguish these types of evidence and compare implementations under explicitly documented workloads.

This work investigates CHIMA (Cryptography cHaotic lIghtweight systeM and Authentication), an experimental composition of a key generator based on chaotic states, a Feistel cipher, and a Lesamnta-LW-based ciphertext check. The Feistel structure, AES S-box, AES-128 key expansion, and hash function are established cryptographic components. The contribution investigated in this work is the particular composition of these components, including the extraction and mixing of chaotic-state bits and their use in the proposed round function. This contribution is an implementation and design proposal; it is not evidence that the individual components, or their composition, provide a new established security guarantee.

This study makes three contributions:

1. It describes the proposed chaotic-based key-generation and cipher constructions, distinguishing the 64-bit and 128-bit block variants and their common 128-bit master key representation.
2. It reports functional checks, diffusion measurements, and NIST statistical tests, while separating these observations from claims of cryptographic security.
3. It measures implementation costs on three Arm microcontroller platforms and demonstrates controlled communication interoperability through WiFi/MQTT and LoRa links.

The measurements identify cases in which the evaluated CHIMA implementation has lower combined encryption/decryption time than selected implementations of PRESENT, AES-128, and CLEFIA. They also show that LEA and the evaluated Ascon implementation are faster than CHIMA-128. The purpose of the comparison is to characterize these measured trade-offs, rather than to claim an established security or efficiency advantage over established cryptographic algorithms.

## 2. Materials and Methods

The proposed symmetric cryptosystem follows the architecture illustrated in Figure 1. The workflow begins with the Chaotic System Computation, which simultaneously drives the 128-bit chaotic key generator and provides the chaotic state used by the integrity-check stage. The generated 128-bit master key is expanded by the AES-128 Key Scheduler into 44 sub-keys of 32 bits each, which supply both the encryption and decryption rounds.

At the transmitter, the Original Data (plaintext) is processed through a Feistel Network incorporating the keyed F Function Permutation. The resulting ciphertext and the chaotic-state value are then processed using the Lesamnta-LW hash function during the Integrity Hash Generation stage to form the transmitted payload (Encrypted Data). This payload is transmitted across the designated communication protocol.

At the receiving end, the incoming Encrypted Data is first evaluated by Integrity Hash Verification using Lesamnta-LW and the synchronized chaotic computation state. Upon successful integrity validation, the ciphertext is decrypted through the receiver’s Feistel Network using the scheduled sub-keys and round permutations to reconstruct the Original Data. Each of these functional components is detailed in the following subsections.

### 2.1. Secret-Key Generation

The proposed method for secret-key generation is based on chaotic systems. The literature presents works that use chaotic systems as key generators in different ways. For a system to be considered chaotic, it must satisfy well-established properties: sensitivity to initial conditions, topological transitivity, and the presence of dense periodic orbits. This section discusses only the characteristics of chaotic systems relevant to this work. Further details can be found in [17]. Chaotic systems are often computed iteratively from a known equation and an initial condition. Their sensitivity to initial conditions makes them potentially useful for pseudorandom number generation. With a slight modification of the initial condition and the same number of iterations, the system has a different behavior, and consequently different output values. On the other hand, if the initial condition and the number of iterations remain the same, the system consistently provides the same result, demonstrating its deterministic nature [13,14,15].

For secret-key generation, three chaotic systems were chosen: the logistic map, the Hénon map, and the Lorenz system. Therefore, the three systems were evaluated in terms of their computational cost and key-generation time. One initial hypothesis is that a more complex chaotic system may provide more complex key material at the cost of increased key-generation time. However, as presented in the results followed by the discussion, this is not necessarily true. The logistic map is computed by Equation (1).

(1) $$x_{n+1}=rx_{n}\left(1-x_{n}\right)$$

Each new value $x_{n+1}$ is computed from the previous value $x_{n}$. The state of *x* evolves through repeated iterations of the map. With the variation of the *r* parameter, orbits of different lengths appear in *x*. To perform this analysis, Figure 2 presents the bifurcation diagram of the logistic map. As the value of *r* increases, the result *x* shows different behaviors. For example, in the interval 3.2<r<3.4 the system exhibits a period-two orbit, and with r=4, the system exhibits the well-known chaotic regime of the logistic map. The use of chaotic regions to generate secret keys was used here, since it is only possible to rebuild the secret key when the same secret key can be reconstructed only when the same initial condition $x_{0}$, parameter *r*, and number of iterations are used.

The Hénon map is a two-dimensional system represented by Equation (2).

(2) $$\begin{array}{cc} x_{n+1} & =y_{n}+1-ax_{n}^{2}\\ y_{n+1} & =bx_{n} \end{array}$$

The iterations of the variables *x* and *y* are given by coupled equations with *a* and *b* as parameters. Depending on the choice for *a* and *b*, the system may exhibit chaotic behavior. Figure 3 presents an example of a bifurcation diagram of the Hénon map with the variation of the parameter *a*. The diagram reveals regions of both order and chaotic behavior. For secret-key generation, the parameter *a* should be selected within a region exhibiting chaotic behavior. The Hénon map is a more elaborate system than the logistic map, as it involves two equations and two parameters.

The Lorenz system is defined by three coupled differential equations, which are discretized here as given in Equation (3).

(3) $$\begin{array}{cc} x_{n+1} & =x_{n}+\sigma \left(y_{n}-x_{n}\right)\Delta t\\ y_{n+1} & =y_{n}+\left(x_{n}\left(\rho -z_{n}\right)-y_{n}\right)\Delta t\\ z_{n+1} & =z_{n}+\left(x_{n}y_{n}-\beta z_{n}\right)\Delta t \end{array}$$

Here, the iterations of variables *x*, *y*, and *z* are given by the coupled equations with σ, ρ and β as parameters and Δt as the time evolution. This chaotic system is more elaborate than the Hénon map and the logistic map because it involves three coupled equations and three parameters. Figure 4 presents an example of a bifurcation diagram for the Lorenz system considering *x* and the variation of the parameter ρ. Again, the parameters should be chosen considering the chaotic regions of the system. In this sense, the Lorenz system is more complex and, therefore, could provide more complex secret keys; however, the computation time is higher. Again, this assumption is not necessarily true, as discussed in the Section 4.

The first step in generating the chaotic key is to select one of the three chaotic systems, the parameters, and the number of iterations. The number of iterations must be large enough for the system to reach the desired chaotic regime. After the iterations are completed, the *x* values from the last eight iterations are combined to form the master key *K*. Each value *x* is converted into a binary representation using the IEEE 754 standard, with rounding to the nearest mode. The value of *x* is composed of 32 bits; the 16 most significant bits (MSBs) are discarded. The least significant bits (LSBs) of the *x* values are concatenated to represent *K*. Let $\left\{x_{1},x_{2},\ldots ,x_{8}\right\}$ be the last 8 *x* iteration values of a given chaotic system, where $x_{1}$ is the last iteration value. Then the values of the iteration results are concatenated in pairs, i.e., $x_{1}⨆x_{2},x_{3}⨆x_{4},x_{5}⨆x_{6},x_{7}⨆x_{8}$, where ⨆ represents the concatenation operation. For each concatenation pair, an avalanche function *A* given by Equation (4) is applied, where α is a fixed mixing constant, ⊕ is the xor operation, and >>16 is a 16-bit shift operation.

(4) A(x)=(αx)⊕((αx)>>16)

Finally, the master key *K* is given by Equation (5).

(5) $$K=A\left(x_{1}⨆x_{2}\right)⨆A\left(x_{3}⨆x_{4}\right)⨆A\left(x_{5}⨆x_{6}\right)⨆A\left(x_{7}⨆x_{8}\right)$$

The representation in Equation (5) contains 128 output bits. This output length is not an estimate of entropy or an established 128-bit security level. The generator is deterministic: its unpredictability is bounded by the uncertainty in its secret inputs and by any structure or collisions introduced by finite-precision iteration and output extraction. A fixed multiplier, bit mixing, and AES-128 key expansion cannot create entropy that is absent from the input. The AES key schedule expands the representation into 44 words of 32 bits for use by the cipher; it does not establish the security of the underlying seed-generation process [48].

Finite-precision chaotic implementations have a finite state space. Their trajectories may enter cycles, and the usable seed space depends on the exact representation, parameter restrictions, and sampling procedure. Sensitivity observed in real-valued dynamical systems therefore does not by itself establish cryptographic unpredictability for the implemented generator. Reproducibility additionally requires the numerical format, evaluation order, rounding behavior, and treatment of intermediate results to be specified. To the best of the authors’ knowledge, this key-generation method is novel in the existing literature.

### 2.2. Encryption and Decryption

Since the proposed method follows the symmetric approach, the Feistel network was chosen for the encryption and decryption scheme. The Feistel network is a widely used construction for block ciphers with the advantage of using the same structure for encryption and decryption with the same cryptographic function *F*. Figure 5 presents an example of the Feistel network. Typically, the Feistel network uses 64 bits as a block size. Then these blocks are divided into left $L_{i}$ and right $R_{i}$ halves, with 32 bits each. Note that the right-hand block $R_{i}$ is copied to the left-hand block $L_{i+1}$ in the next round. The right-hand block $R_{i}$ is used as input to the encryption function *F* and then an XOR operation is performed with the left block $L_{i}$ to provide the right block $R_{i+1}$ of the next round. Finally, the blocks $L_{i+1}$ and $R_{i+1}$ are concatenated to form the encrypted data.

This operation is executed *N* times during encryption and *N* times during decryption. The advantage of using this approach is that the structure and the function *F* are the same for both processes and that *F* does not need to be reversible. Here, the Feistel network can be described by Equation (6).

(6) $$\begin{array}{cc} L_{i+1} & =R_{i}\\ R_{i+1} & =L_{i}⊕F\left(R_{i},\left\{k_{2m},k_{2m+1}\right\}\right) \end{array}$$

In the method presented here, the set of keys $K=\{k_{2m},k_{2m+1}\}$ for each round has the index range m={0,1,2,…,M}, with *M* depending on *N*. *N* is limited to the number of round keys. For example, if each round uses 2 round keys of the 44 keys of 32 bits available, then the maximum number of rounds in this case is N=22.

Taking into account that the Feistel network was developed to work with block sizes of 64 bits, here it was decided that for the 128-bit version the two 32-bit round keys are concatenated to form a single 64-bit value, which is then used for both key inputs of the function *F*. In other words, for the 128-bit version, the input block sizes (left and right hands) were extended to 64 bits. Note that the round keys must also be taken in 64 bit, i.e., concatenate two 32-bit keys; in this case the two round keys of the function *F* are the same and given by $k_{2m}⨆k_{2m+1}$ and the number of rounds N=22.

The cryptographic function *F* is presented in Figure 6. First, the right-hand block $R_{i}$ is used as input, and an xor ⊕ operation is performed with the key $k_{2m}$. Then, a substitution in the S-box of the AES-128 follows, namely operation S(·). Finally, the permutation operation P(·) proposed by [15] is applied using the key $K_{2m+1}$. The cryptography function for the 64-bit version is described in Equation (7) and for 128-bit version given by Equation (8).

(7) $$F\left(R_{i},\left\{k_{2m},k_{2m+1}\right\}\right)=P\left(S\left(R_{i}⊕k_{2m}\right),k_{2m+1}\right)$$

(8) $$\begin{array}{cc} \; & F(R_{i},\left\{k_{2m},k_{2m+1}\right\})=\\ \; & P(S\left(R_{i}⊕\left(k_{2m}⨆k_{2m+1}\right)\right),\left(k_{2m}⨆k_{2m+1}\right)) \end{array}$$

In order to clarify the usage of the proposed *F* function, an example of 8 bits is presented for the first round. Let $R_{0}=10100101$ be the input of the function together with the keys $k_{0}=00111100$ and $k_{1}=10110010$. First, the operation $R_{0}⊕k_{0}$ is computed, resulting in 10011001 or 0x99 in hexadecimal representation. Then, substitution is performed using the AES S-box. The hexadecimal value 0x99 corresponds to row 9 and column 9 of the AES S-box presented in Table 1, yielding 0xEE or 11101110 in binary representation, now namely data *D*.

The AES substitution box was selected as an existing, precisely specified 8-bit nonlinear substitution [48]. In CHIMA, it is applied to each byte of the half-block after the round-key XOR. This choice avoids introducing an additional, unspecified substitution table and allows a direct lookup-table implementation. The single-byte AES S-box contains 256 entries, although its actual memory and timing costs depend on the implementation and target architecture. These costs must be measured rather than inferred from the use of an AES component.

Reusing the AES substitution box does not transfer the security of AES to CHIMA. AES combines its substitution with a specific linear layer, round structure, and key schedule; CHIMA uses a different Feistel construction and a keyed bit permutation. The composition must therefore be analyzed on its own. A bit permutation relocates bits without changing their values and does not, by itself, increase the number of differing bits in a fixed-key difference. Its contribution to multiround diffusion depends on the surrounding substitutions and Feistel rounds. Lookup-table accesses and key-dependent operations also require implementation-specific side-channel evaluation before claims of constant-time behavior can be made.

The last step is the permutation operation, presented in Figure 7. The process occurs from right to left. Following the example, the first bit of $k_{1}$ is a 0, then the first output bit of $P(D,k_{1})$ is the first bit of *D*. The next bit of $k_{1}$ is one, then the next bit of $P(D,k_{1})$ is the last available bit of *D*. Then, the next bit of $k_{1}$ is a zero again, and then the next bit of $P(D,k_{1})$ is the next available bit of *D*, moving from right to left. This process continues until the end of $k_{1}$.

#### Explicit Bit-Order Convention for the Permutation

To make the bit ordering explicit, please refer to Algorithm 1. Let D[j] and Q[j] denote bit *j* of the data and the permutation key, with j=0 corresponding to the least significant, rightmost bit. The following specification fills output bits in increasing index order. A zero key bit selects the lowest-index unused input bit, and a one key bit selects the highest-index unused input bit.
**Algorithm 1** Bit permutation algorithm**Require:** *w*-bit values *D* and *Q*  1:lo←0, hi←w−1, Y←0  2:**for** j←0 to w−1 **do**  3:      **if** Q[j]=0 **then**  4:            Y[j]←D[lo]  5:            lo←lo+1  6:      **else**  7:            Y[j]←D[hi]  8:            hi←hi−1  9:      **end if**10:**end for**11:**return** *Y*

For each fixed *Q*, every bit of *D* is selected exactly once. Consequently, this operation is a permutation of the input bits. When only one input bit remains, either key-bit value selects the same bit, so distinct permutation keys can describe the same permutation. This observation should be considered when analyzing effective round-function key dependence; it is not by itself a demonstrated attack on the complete cipher.

### 2.3. Ciphertext Check in the Evaluated Prototype

The evaluated prototype computes, the evaluated prototype computes a Lesamnta-LW hash over the ciphertext concatenated with a shared value obtained from the final chaotic iteration. Let *C* denote the ciphertext, *s* the shared value, and *H* the hash function. The transmitted check is

(9) T=H(C∥s).

The receiver recomputes Equation (9), compares the result with the received *T*, and proceeds to decryption only if the values agree. This check concerns transmitted messages; it is not a user-authentication protocol and does not establish the identity of a human user.

The construction in Equation (9) is not HMAC. HMAC uses a prescribed nested keyed construction with inner and outer padding, as defined in RFC 2104 [49]. If *s* is public, any party can compute a check for a modified ciphertext. If *s* is secret, Equation (9) is an ad hoc keyed construction whose security requires separate justification; the collision or preimage resistance of the unkeyed hash alone does not supply that justification.

If the shared value *s* is serialized from the same binary32 state representation described for key extraction, it has at most $2^{32}$ possible bit strings. If that suffix is the only secret required to compute the check, an observed ciphertext–check pair permits offline testing of candidate suffixes. Exhaustive enumeration then requires at most $2^{32}$ hash evaluations, with potentially fewer candidates when valid floating-point values or parameter restrictions reduce the set. This is a conditional bound on the described check, not a measured attack or an estimate of the complete cipher’s security. It also shows why the nominal 128-bit master key representation cannot be used to claim 128-bit authentication strength for this construction.

The described check has no freshness field or replay rejection rule. An unchanged ciphertext–check pair may therefore be accepted again unless the surrounding protocol supplies separately specified and authenticated replay protection. A matching check alone does not distinguish a fresh message from a retransmission and does not demonstrate general protection against a man-in-the-middle adversary. The communication experiments assess the behavior of the prototype under the tested conditions, rather than adversarial message-authentication security.

### 2.4. Proposed Threat Model and Protocol Requirements

The following threat model is proposed to guide a subsequent security evaluation. It is not a claim that the current prototype meets the listed requirements. The adversary is assumed to know the algorithm and implementation specification and to observe, intercept, modify, inject, reorder, and replay network messages. The adversary may also obtain ciphertexts for selected plaintexts and submit modified ciphertexts to the receiver. Initial secret provisioning is assumed to occur through a trusted mechanism, but such a mechanism and its lifecycle are not specified or evaluated in the present implementation. Physical extraction of secrets, fault injection, and side-channel leakage remain outside the current experimental scope and must not be interpreted as attacks against which the prototype has been shown to provide resistance.

Under this model, a deployable design would need authenticated provisioning or key establishment, an explicit separation between secret inputs and public parameters, protected secret storage, defined key rotation and compromise recovery, and authenticated freshness information. Encryption keys and authentication keys would require appropriate separation. Received messages would need an unambiguous encoding that binds the ciphertext to its protocol context, device or session identifier, and freshness information; failed verification would have to prevent release of plaintext. These are requirements for a revised protocol, not features measured by the current benchmarks.

A revision could evaluate a standardized authenticated-encryption construction, such as Ascon-AEAD128 from NIST SP 800-232 [50], as an end-to-end reference. Alternatively, retaining an experimental cipher would require a fully specified and justified encryption-and-MAC composition, independent key material, and a standard MAC such as HMAC [49]. Either change would define a different implementation from the current suffix-hash prototype and would require new functional tests and performance measurements. Authenticating only a handshake would not authenticate subsequent application messages by itself.

## 3. Experimental Methodology

The evaluation distinguishes three questions. Functional tests examine whether encryption and decryption conform to the proposed specification and recover the intended plaintext. Avalanche measurements examine the propagation of controlled input changes. NIST SP 800-22 tests examine selected statistical properties of generated bit sequences [47]. None of these tests, individually or together, establishes resistance to cryptanalysis, predicts the entropy of undisclosed secret seeds, or certifies CHIMA under FIPS 140-3. FIPS 197 specifies AES and is used here to identify the reused AES components, not as a validation standard for the new cipher [48].

For a statistical test, a *p*-value above the selected significance threshold means that the corresponding null hypothesis was not rejected for the tested sequence. It is not the probability that the sequence is random and does not quantify cryptographic security. Results should report all applicable subtests, the number of eligible sequences, pass proportions and their acceptance intervals, and the prescribed assessment of *p*-value uniformity. Mean or maximum *p*-values cannot replace these checks, and a large maximum does not establish that the remaining sequences passed. Eligibility restrictions for tests such as random excursions must also be reported [47].

### 3.1. Statistical Evaluation of Chaotic Key Generation

We evaluated the three options of chaotic systems for the generation of master keys. The NIST Statistical Test Suite (STS) was used to evaluate the statistical properties of the generated key sequences. The NIST Statistical Test Suite (STS) includes 15 statistical tests: approximate entropy, block frequency, cumulative sums, fast Fourier transform (FFT), frequency, linear complexity, longest run, nonoverlapping template, overlapping template, random excursions, random excursions variant, rank, runs, serial and universal.

The NIST tests require sequences of 1,000,000 bits for the master key evaluation; thus, for each test round 8000 keys of 128 bits were used. The NIST standard recommends 10 rounds with different keys; therefore, 80,000 keys were used for a balanced analysis. NIST tests are based on distributions such as standard normal, chi-square distribution, binomial distribution, and other statistics derived from the expected behavior of random sequences. Each test in the suite uses one of these distributions to calculate the *p*-value and assess whether the sequence being analyzed can be considered random. For a sequence to pass an individual test, the resulting *p*-value must exceed the selected significance threshold of 0.01.

The evaluation considered testing procedures and references commonly used in cryptographic implementations, including FIPS 197 [48], NIST SP 800-22 [47], and NIST SP 800-38A [51]. FIPS 140-3 [52] was considered as a reference for the broader requirements applicable to cryptographic modules, but no FIPS 140-3 validation was performed.

### 3.2. Cipher Validation: Diffusion, Functional, and Statistical Tests

The first evaluation was the avalanche effect test, which examines whether small changes in the input propagate to substantial changes in the output, resulting in changes distributed across the output bits. This behavior is an important indicator of diffusion in a block-cipher construction. A secure algorithm must be highly sensitive to any minor modification of the plaintext or key, so that the ciphertext changes in a widely distributed manner.

Nine distinct categories of test vectors were generated to explore different aspects of diffusion and sensitivity: avalanche key, avalanche plaintext, correlation, CBC (Cipher Block Chaining), random plaintext and key (PT/Key), low-density plaintext, low-density key, high-density plaintext and high-density key. For each category, 384 sequences were generated, each with 1,048,576 bits (equivalent to 128 KiB of data), totaling more than 350 MiB per category. The tests were performed with 64-bit (8-byte) and 128-bit (16-byte) blocks. The algorithm was tested using all traditional modes (Electronic Codebook—ECB; Cipher Block Chaining—CBC; Cipher Feedback—CFB; Output Feedback—OFB; Counter—CTR); however, for the paper organization, only the ECB results are presented, and the other results are discussed. The results were recorded in files following the standard test vectors used in the NIST validations. For each pair of consecutive cipher blocks, the Hamming distance was calculated, allowing evaluation of the bit-by-bit variation between the outputs. These tests provide an initial functional and diffusion assessment of CHIMA.

The functional tests were then computed, in which the aim is to ensure correct encryption, decryption, reversibility, and proper behavior in different operating modes. The Known Answer Test (KAT) aims to validate the accuracy of the cryptographic algorithm by comparing encrypted outputs with previously known reference values (standard vectors). The test consists of comparing the output produced by the algorithm bit by bit with the expected value. Verification was performed for both the encryption and decryption of the blocks, ensuring reversibility. The Monte Carlo Test (MCT) evaluates the stability and reversibility of the algorithm after a long sequence of consecutive encryptions. For each test vector, 100 cycles with 1000 consecutive iterations were performed. The cipher output in each iteration was used as input for the next iteration, forming a dependency chain. In each cycle, the key and the plaintext were updated with XOR operations, as defined in the MCT algorithm. The reversibility of the process was confirmed by fully decrypting the final output and comparing it with the original data. Binary consistency was required between the data after cascading encryption and decryption. The Multi-Block Message Testing (MMT) verifies the cipher’s behavior with long messages composed of multiple blocks, simulating the real use of the algorithm in continuous data streams. Input files with hexadecimal keys, variable-length messages, and initialization vectors were used. The messages were segmented into 128-bit blocks and encrypted block by block according to the selected operating mode. After decryption, the data were fully compared with the original values to confirm integrity and reversibility. For each implemented operating mode (ECB, CBC, CFB, OFB, CTR), a set of tests was conducted with the following objectives: Correctly encrypt and decrypt data blocks, keep distinct behavior between modes, even with the same input, confirm complete reversibility of the process, validate the correct use of initialization vectors and counters when applicable, and check the sensitivity of the modes to variations in the input. These functional tests ensure that the CHIMA algorithm operates correctly under standardized conditions, providing a foundation for subsequent phases of statistical validation and security evaluation.

The statistical tests were used to characterize the output sequences of CHIMA under the specified test conditions. Passing these tests indicates that the tested sequences do not exhibit the statistical properties targeted by the corresponding tests; it does not establish entropy, unpredictability, or cryptographic security. Compliance with the NIST SP 800-22 test suite is a key indication that the cipher has characteristics of high entropy and unpredictability, even with similar or structured inputs. Samples of cipher blocks with 1,048,576 bits (128 KiB) were generated in each of the following categories: avalanche key, avalanche plaintext, correlation, CBC (Cipher Block Chaining), random plaintext and key (PT/Key), low-density plaintext, low-density key, high-density plaintext, and high-density key. Each category represents a specific test scenario, simulating the adverse or realistic conditions that an algorithm might face in practical applications. And again, the same 15 official tests of the NIST STS suite used for the key generator were applied here.

### 3.3. Embedded Hardware Implementation and Benchmark Baselines

We also implemented all algorithms on different microcontrollers, using Arm Cortex-M0+, Cortex-M33, and Cortex-M7 microcontrollers from STMicroelectronics:

- NUCLEO-G0B1RE: Arm 32-bit Cortex-M0+ (part number STM32G0B1RET6U), clock frequency up to 64 MHz, and 144 Kbytes of SRAM with no floating-point unit;
- NUCLEO-H533RE: Arm 32-bit Cortex-M33 (part number STM32H533RET6U), clock frequency up to 250 MHz, and 272 Kbytes of SRAM with hardware single-precision floating-point unit;
- NUCLEO-H755ZI: Arm 32-bit Cortex-M7 (part number STM32H755ZIT6U), clock frequency up to 480 MHz, and 1060 Kbytes of RAM with hardware double-precision floating-point unit.

The energy consumption and execution time of each algorithm were measured. For energy consumption, the STLINK-V3PWR from STMicroelectronics was used, which provides debugging capabilities and current-consumption measurements.

In order to evaluate whether the results are consistent, the results of the algorithm proposed here were compared with other established cryptographic algorithms. The chosen benchmarks were those cited in ISO/IEC 29192-2:2012 [53], PRESENT [54], LEA [55], and CLEFIA [56], which are well-established lightweight cryptographic algorithms. We also compared the proposed algorithm with ASCON [57] from NIST and finally with AES [58].

### 3.4. Communication and Interoperability Testbed

Finally, the algorithm was implemented on microcontrollers and encrypted messages were transmitted using different communication technologies to evaluate a real application. Wi-Fi/MQTT and point-to-point LoRa communication were used to exchange cryptographic messages using the following devices:

- SparkFun Things Plus-ESP32-S2 WROOM: Development Board equipped with a 2.4 GHz WiFi System-on-Chip (SoC) from Espressif, Xtensa Single-Core 32-bit LX7 Microprocessor (up to 240 MHz), RISC-V ULP Coprocessor, 128 KB ROM, 320 KB SRAM and 4 MB of Embedded SPI Flash Storage;
- SparkFun LoRaSerial Kit: Long-range serial radio modem, 915 MHz frequency band, 1 W (30 dBm) power output, 15 km range line of sight, and 300 m urban range.

These experiments evaluated the feasibility of using the proposed algorithm to encrypt the payload of the aforementioned protocols. In addition, it was possible to test the encryption algorithm in one device and the decryption in another device. Figure 8 presents an example of one test. Three microcontrollers were tested with the two transmission technologies and with two versions (64 and 128 bit) of CHIMA.

In the reported communication setup, the STM32 devices perform the cryptographic operations, while the ESP32-S2 boards provide serial-to-Wi-Fi/MQTT connectivity. This allocation was adopted to separate the communication/connectivity functions from the cryptographic processing performed on the STM32 devices. Therefore, the ESP32-S2 was not used to execute CHIMA in the reported experiments. This design choice does not imply that the ESP32-S2 is unable to run CHIMA; rather, it was made to ensure a clear separation between the communication and cryptographic processing functions.

## 4. Results

The experiments are organized into three scenarios. Scenario A evaluates chaotic-state-derived key generation on the Cortex-M0+, Cortex-M33, and Cortex-M7 platforms. Scenario B evaluates cipher processing and memory use on the same platforms. Scenario C evaluates communication interoperability by encrypting a message on one endpoint, transmitting the ciphertext through Wi-Fi/MQTT or LoRa, and recovering the message on another endpoint. This separation distinguishes the costs of primitive operations from the behavior of the complete communication path.

### 4.1. Scenario A: Chaotic Secret-Key Generation

The proposed chaotic secret-key generator was tested using three different chaotic systems: the logistic map, the Hénon map, and the Lorenz system. The tests followed the NIST Statistical Test Suite (STS) for Random and Pseudorandom Number Generators for Cryptographic Applications. As described in Section 3, for each test, a sequence set was considered to meet the selected acceptance criterion when at least eight of 10 sequences yielded *p*-values above 0.01. The NIST test results are presented graphically for clarity.

The first test was performed with the logistic map. The value α=0xd168aaad was used for all tests. Figure 9 presents the results of the NIST tests considering the logistic map. To generate 80,000 master keys, the logistic map was iterated 1000 times for each initial condition chosen in the interval $0.01\leq x_{0}<1$ and for the parameter *r* in the interval 3.9≤r<4. The mean, standard deviation, median, and maximum *p*-values are reported to summarize the test results. The reported mean and maximum *p*-values provide a summary of the observed results; the individual test outcomes and pass proportions are required to assess the selected acceptance criterion. The median and standard deviation are also reported to characterize the distribution of the observed *p*-values.

The second test was performed using the Hénon map with initial conditions $0.02\leq x_{0}<1.02$, $0.02\leq y_{0}<0.2$, parameters 1.4≤a<1.41 and b=0.3, and 1000 iterations to generate the 80,000 master keys. Figure 10 presents the results for the Hénon map in the generation of master keys. Note again that the mean and highest values are greater than 0.01.

Finally, the Lorenz system with an initial condition range of $1\leq x_{0}<1.5$, $1\leq y_{0}<1.3$, $1.05\leq z_{0}<1.25$, parameters σ=10, $\beta =\frac{10}{3}$ and 28≤ρ<30, dt=0.01 and 1000 iterations was tested to generate the 80,000 master keys. Figure 11 presents the results for the generation of master keys from the Lorenz system. The mean and highest values are higher than 0.01, confirming the approval of the generator in the NIST statistical standard tests, with good results for the median and standard deviation, as well.

To analyze the computational overhead of the candidate chaotic dynamics, an isolated algorithmic microbenchmark was first conducted to measure 1000 iterations of each map within a standalone test harness. For each platform, each system was computed five times, and the results are summarized in Table 2. As expected, the logistic map required a significantly lower computation time than the higher-dimensional systems across all evaluated targets. Because all three maps met the selected NIST SP 800-22 statistical criteria under the tested conditions, the additional computational cost of the higher-dimensional systems did not provide a corresponding benefit under the evaluated criteria in resource-constrained IoT nodes. Consequently, the logistic map was adopted as the baseline generator for the remainder of this work.

Following this selection, Table 3 and Table 4 present the execution time and energy consumption, respectively, of the complete 128-bit master key-generation routine integrated into the final firmware stack (combining the 1000 logistic map iterations and the post-processing avalanche function A(x)). It is worth noting that minor execution differences between the standalone map benchmark (Table 2) and the integrated driver routine (Table 3)—such as the approximately 1.9% timing difference observed on the Cortex-M0+—are influenced by factors such as instruction caching/prefetch behavior, Flash memory alignment, and loop encapsulation overhead inherent to production-level drivers compared to an isolated benchmark harness. Furthermore, because master key derivation is executed exclusively during initial session provisioning or scheduled rekeying windows, this latency does not affect real-time, per-packet telemetry processing.

### 4.2. Scenario B: Cipher Performance and Benchmarking

This scenario evaluates the implementation of the CHIMA cipher across the target Arm Cortex-M platforms in comparison with the established baseline ciphers. The evaluation addresses round-tuning and functional validation, processing time for encryption and decryption, energy consumption per block, and memory footprint (RAM and Flash utilization).

#### 4.2.1. Cryptographic Evaluation

The two versions of CHIMA were tested with rounds in the range 1<N<22. The algorithm presented satisfactory results with N=9 rounds in both versions (64 and 128 bits). For N<9, the implementation did not meet the selected test criteria, while increasing *N* beyond 9 did not produce a significant improvement in the evaluated metrics. As expected, increasing the number of rounds increases computational cost without a measurable benefit under the evaluated criteria. Therefore, all the results presented here refer to the 64-bit and 128-bit versions of CHIMA with N=9 rounds. Figure 12 and Figure 13 present the results of the avalanche tests for the 64-bit and 128-bit versions of CHIMA, respectively. Note that for all boxplots the distributions are centered close to 50%.

The CHIMA algorithm was then tested following functional tests. Functional tests were described in Section 3 and applied in the two versions of CHIMA. The tests produce binary pass/fail outcomes. Table 5 shows that CHIMA passed all functional tests.

Finally, statistical tests were applied to CHIMA in both versions. Figure 14 presents the CHIMA results for the statistical tests. For compact presentation, Figure 14 reports the maximum *p*-value observed for each test. The results indicate that the tested output sequences did not exhibit the statistical properties targeted by the applied NIST tests under the evaluated conditions.

The functional, diffusion, and statistical evaluations produced the following observations, of which the following stand out: effective avalanche effect, with high propagation of changes in the output, reversibility, and consistency in all operating modes, correct response to Known Answer Test vectors, and consistent statistical behavior under the applied tests, all validated by the NIST STS suite.

#### 4.2.2. Time for Encryption and Decryption

Encryption and decryption times were measured on the three devices. The proposed algorithm was compared with PRESENT in case of 64 bits and with LEA, CLEFIA, AES, and ASCON in case of 128 bits. Table 6 presents the comparison of the proposed algorithm with PRESENT 64 considering time consumption and the three devices. In general, CHIMA-64 required approximately 64% less combined encryption/decryption time than PRESENT. Note that, as expected, the M7 is much faster than the other two devices.

Table 7 presents the time consumption comparison of the 128-bit algorithms. The time performance of CHIMA 128 was better than that of CLEFIA 128 and AES 128, and worse than that of ASCON 128 and LEA 128 considering the summing of encryption and decryption times. LEA-128 had the lowest combined encryption/decryption time, whereas AES-128 had the highest among the evaluated implementations. Note again the better time performance in the M7 device for all algorithms.

For a descriptive comparison of the existing measurements, define the combined processing time as $T_{\mathrm{pair}}=T_{\mathrm{enc}}+T_{\mathrm{dec}}$. Relative reduction with respect to a reference implementation is computed as

(10) $$\Delta_{T}=100\left(1-\frac{T_{\mathrm{pair},\mathrm{CHIMA}}}{T_{\mathrm{pair},\mathrm{reference}}}\right)\%.$$

This metric assigns equal weight to one reported encryption measurement and one reported decryption measurement. It is not a complete communication latency, and its relevance depends on the application’s encryption/decryption workload and the measurement scope.

Using the values in Table 6, CHIMA-64 has a lower combined time than PRESENT by 63.08%, 64.16%, and 57.95% on the Cortex-M0+, Cortex-M33, and Cortex-M7, respectively. Using Table 7, the corresponding reductions for CHIMA-128 relative to AES-128 are 23.60%, 39.74%, and 59.36%, and those relative to CLEFIA are 13.32%, 29.18%, and 47.10%.

These combined values should be interpreted alongside the individual operation times. The reported AES implementation encrypts faster than CHIMA-128 on all three platforms; its larger decryption times account for the resulting comparison under $T_{\mathrm{pair}}$. LEA and the evaluated Ascon implementation have lower combined times than CHIMA-128 on all three platforms. The Cortex-M7 is also not uniformly the fastest platform: the reported LEA, Ascon, CLEFIA, and AES values include operations that are faster on the Cortex-M33. These results describe the particular implementations and configurations evaluated.

#### 4.2.3. Energy Consumption for Encryption and Decryption

The energy consumption in each device was measured using the STLINK-V3PWR and STM32CubeMonitor-Power. The sampling frequency was 100,000 Hz and the total acquisition time was 100 s. Table 8 presents the comparison of the energy consumption of CHIMA 64 and PRESENT 64 for the three devices. CHIMA-64 consumed approximately 61% less energy than PRESENT-64.

Table 9 presents the energy consumption measurements of the 128-bit algorithms. CHIMA-128 consumed less energy than CLEFIA-128 and AES-128 and more energy than LEA-128 and ASCON-128, considering the sum of encryption and decryption energy consumption. LEA-128 had the lowest combined energy consumption, whereas AES-128 had the highest among the evaluated implementations. Considering the devices in both Table 8 and Table 9, one might note that the device M33 has a lower energy consumption than M7. As shown in Table 7, the LEA-128 and ASCON-128 execution times are lower on the Cortex-M33 than on the Cortex-M7.

The observed differences between the Cortex-M33 and Cortex-M7 results may depend on clock configuration, compiler-generated instructions, memory placement, cache behavior, peripheral activity, and the measurement boundary. The present measurements do not isolate these factors. Architectural attribution therefore requires targeted profiling and controlled configurations; it cannot be established solely from the processor family names or nominal maximum clock frequencies.

These results highlight the importance of evaluating cryptographic implementations on the target architecture rather than extrapolating performance from nominal processor specifications. The STM32H755 is a dual-core microcontroller, although only its Cortex-M7 core was used in these experiments. The measured energy consumption therefore reflects the complete experimental hardware configuration and measurement boundary, rather than the active core alone.

#### 4.2.4. Memory Usage

The memory footprints compiled for each cryptographic algorithm across the three target microcontrollers are detailed in Table 10. For CHIMA, memory consumption is reported jointly for both the 64-bit and 128-bit variants because they share the underlying functional implementation within a single unified firmware module—specifically the Feistel network architecture, the AES S-box lookup table, the keyed bit-permutation engine, the chaotic key generator, and the Lesamnta-LW ciphertext verification routine. This design choice prioritizes code modularity and multi-variant flexibility in embedded deployments.

As observed in Table 10, the reported Flash occupancy for the integrated CHIMA stack ranges from 27.81 KB on the Cortex-M0+ to 39.34 KB on the Cortex-M33, while static RAM requires between 2.49 KB and 2.62 KB across the evaluated targets. It is important to emphasize that these build-derived metrics reflect the total memory footprint of the compiled microcontroller firmware image—encompassing the hardware abstraction layer (HAL), system startup code, interrupt vector tables, and peripheral runtime control—rather than the cryptographic routines in complete isolation. Consequently, the memory variations observed across the three platforms stem not only from the cipher implementation itself, but also from target-specific compiler code generation, architectural instruction sets, and the differing baseline overheads of the respective device driver packages. Although this unified binary exhibits a higher overall Flash requirement than standalone, single-purpose ciphers (such as PRESENT-64 or LEA-128), this footprint directly accounts for bundling both 64-bit and 128-bit block-size modes, the chaotic master key generator, and the Lesamnta-LW ciphertext check into a single deployable image. Furthermore, the total static RAM utilization represents less than 1.8% of the available SRAM on the most constrained platform (STM32G0B1RE with 144 KB) and negligible fractions on the higher-end devices, confirming that the entire CHIMA cryptographic and verification pipeline operates comfortably within the operational constraints of edge IoT hardware.

### 4.3. Scenario C: End-to-End Communication and Interoperability

To evaluate the operational feasibility of the CHIMA system under realistic networking conditions, the experimental validation was structured into two clearly defined practical scenarios, separating the evaluation by communication protocol and hardware interfaces:

#### 4.3.1. MQTT over Wi-Fi

In this scenario, communication was evaluated over a standard 2.4 GHz Wi-Fi network connected to a public MQTT broker. The source microcontroller (Cortex-M0+, Cortex-M33, or Cortex-M7) encrypts the message, computes the Lesamnta-LW ciphertext check, and transmits the resulting payload via serial UART to a SparkFun Thing Plus board (ESP32-S2 WROOM). The ESP32-S2 publishes the encrypted data to a designated topic on the public MQTT broker. At the destination, a second ESP32-S2 subscribed to the topic receives the payload and forwards it through serial communication to the target microcontroller, which verifies the integrity check and performs decryption (as illustrated in Figure 8).

#### 4.3.2. LoRa P2P

In this scenario, communication was evaluated over a direct point-to-point (P2P) RF link using the SparkFun LoRaSerial Kit, long-range serial radio modems operating at 915 MHz. The transmitting and receiving microcontrollers were interfaced directly with the LoRaSerial modems via serial UART. This setup enabled the direct point-to-point exchange of encrypted payloads and authentication checks between the target platforms without requiring network-layer protocols.

Across all permutations combining the three target microcontrollers, the two communication protocols, and both algorithm variants (CHIMA-64 and CHIMA-128), messages and ciphertext checks were successfully delivered, verified, and decrypted without observed data loss or synchronization failures. Table 11 presents the time consumption and Table 12 presents the energy consumption of the integrity check on each platform.

These controlled communication experiments demonstrate functional interoperability of the CHIMA prototype across the tested microcontrollers and communication links. The experiments were not designed to evaluate network-level adversarial defenses, such as replay protection or session-state management, nor to characterize field channel conditions. Within the tested configurations, the results show that the implemented encryption, ciphertext check, and decryption operations can be integrated into the evaluated Wi-Fi/MQTT and point-to-point LoRa communication paths.

## 5. Discussion

The study characterizes an experimental cryptographic design and its implementation on three embedded platforms. Its main measured results concern computation time, reported memory use, and communication interoperability. The comparison of chaotic systems identifies the logistic map as the least costly of the three evaluated generators under the reported settings. Similar statistical test results do not establish equivalent seed entropy or resistance to prediction, so this selection reflects an implementation decision rather than a demonstrated security ranking.

The nominal master key representation is 128 bits for both CHIMA variants. The 64-bit and 128-bit names refer to block length, and therefore the latter name does not imply an exponentially larger key space. Effective key-search resistance depends on the entropy and structure of the secret seed and its mapping to the master key. Block length also affects mode-dependent data limits and generic collision effects. Short payloads alone are therefore not sufficient justification for selecting a smaller-block experimental cipher, particularly when authenticated-encryption constructions support variable-length messages.

The nine-round configuration was selected through empirical statistical and diffusion criteria. Neither those criteria nor the reuse of the AES substitution box establishes a security margin. Analysis should address the keyed permutation, dependence between round keys, shared use of sub-keys in the 128-bit round function, finite-precision key generation, and possible differential, linear, structural, or related-key weaknesses under a stated model. Future changes to the S-box, permutation, schedule, or round count would require a revised specification and renewed evaluation rather than being treated as performance-only optimizations.

The current Lesamnta-LW ciphertext–check construction is a separate limitation. It is not HMAC, its effective secret space is constrained by the shared suffix representation, and the described message format does not establish freshness. The observed correct verification of transmitted messages is a functional observation; it does not justify claims of secure user authentication or general resistance to message forgery. A revised protocol should use a justified authentication construction, defined key management, and authenticated freshness information. The cost of that revised protocol must be measured independently of the present prototype.

The timing results show lower combined encryption and decryption times for CHIMA-64 than for PRESENT, and for CHIMA-128 than for the evaluated AES-128 and CLEFIA implementations. However, AES encryption alone is faster than CHIMA-128, while LEA and the evaluated Ascon implementation are faster under the combined metric. The reported CHIMA FLASH footprint is also larger than those of the comparison builds. The available evidence therefore does not support choosing CHIMA over LEA or Ascon on overall performance or established security grounds. Its contribution is the documented exploration of this particular construction and its implementation costs, while identifying the questions that remain unresolved.

ASCON is especially relevant because NIST SP 800-232 specifies Ascon-based authenticated encryption for constrained devices [50]. A fair end-to-end comparison must identify the Ascon variant and give both systems the same functional workload, including authentication, nonce handling, associated data, and comparable message sizes. A raw cipher operation and an authenticated-encryption operation serve different purposes. Similarly, key setup, cipher processing, authentication, communication, and firmware overhead must be separated or consistently included before attributing a measured difference to the primitive itself.

The experimental scope remains limited to selected implementations, three Cortex platforms, and controlled communication tests. The existing point estimates do not quantify confidence in performance differences, and the statistical summaries require complete sequence-level and subtest-level reporting. Releasing the specification, source code, compiler settings, test vectors, raw timing observations, energy traces, and statistical outputs would make the findings reproducible and allow independent verification. Until these methodological and security questions are addressed, CHIMA should be presented as an experimental construction requiring further analysis.

## 6. Conclusions

This work presented CHIMA, an experimental IoT cryptographic system that integrates deterministic key generation based on chaotic states, a Feistel cipher with 64-bit and 128-bit block variants, and a Lesamnta-LW-based ciphertext check. Implementations on Cortex-M0+, Cortex-M33, and Cortex-M7 devices provided an experimental evaluation including functional and statistical analyses, processing-time measurements, and controlled WiFi/MQTT and LoRa interoperability tests. These results show that the proposed cryptographic architecture can be implemented and experimentally evaluated across different resource-constrained embedded platforms and communication environments. The measured combined encryption and decryption times indicate lower processing times for CHIMA-64 compared with PRESENT and for CHIMA-128 compared with the evaluated AES-128 and CLEFIA implementations. LEA and the evaluated Ascon implementation remained faster than CHIMA-128 under the same combined metric, while AES encryption alone was also faster. These results provide a quantitative characterization of the implemented alternatives and characterize the processing performance of CHIMA under the experimental conditions considered in this study. The statistical output behavior and successful decryption tests provided evidence of the functional operation of the prototype, while recognizing that these results alone do not establish cryptographic security. The entropy properties and unpredictability of the generated keys, the round security margin, and the authentication properties of the prototype remain important aspects for further investigation. In this context, the main contribution of this work is the design, implementation, and experimental evaluation of an integrated cryptographic architecture for IoT environments. Further development and validation of CHIMA should include a complete specification, reproducible measurements, independent cryptanalysis, explicit key management, and a justified authenticated-encryption or encryption-and-MAC protocol before considering deployment in practical systems. As part of our future work, we plan to extend the experimental validation to more representative operating environments, involving a broader range of embedded devices, communication protocols, and operating conditions. These experiments will provide a broader assessment of the system and contribute to evaluating its suitability for practical IoT applications.

## Figures and Tables

**Figure 1:**
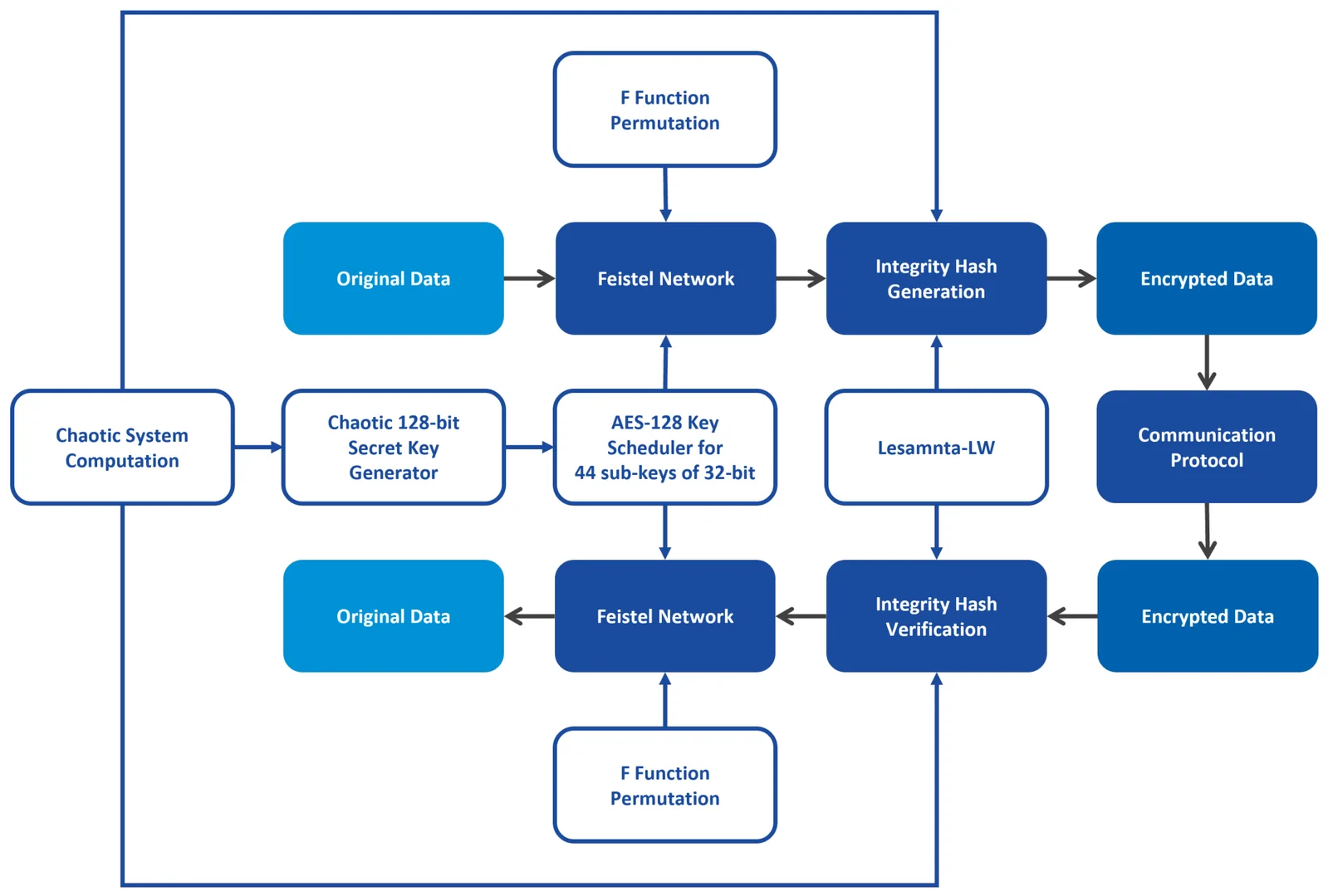
Block diagram with the main parts of the proposed algorithm.

**Figure 2:**
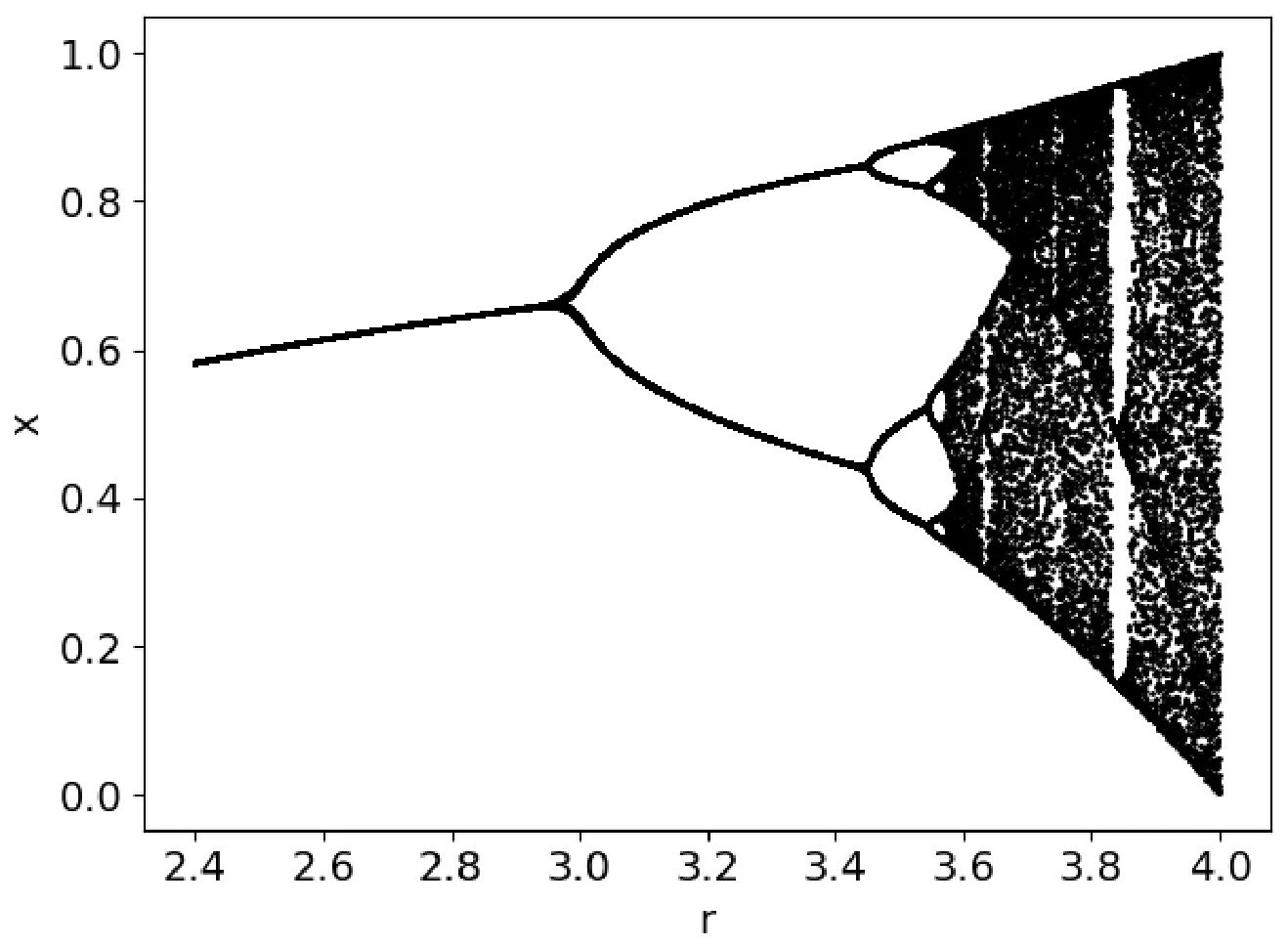
Bifurcation diagram of the logistic map with the initial condition $x_{0}=0.2$ and 100 of iterations for each *r*.

**Figure 3:**
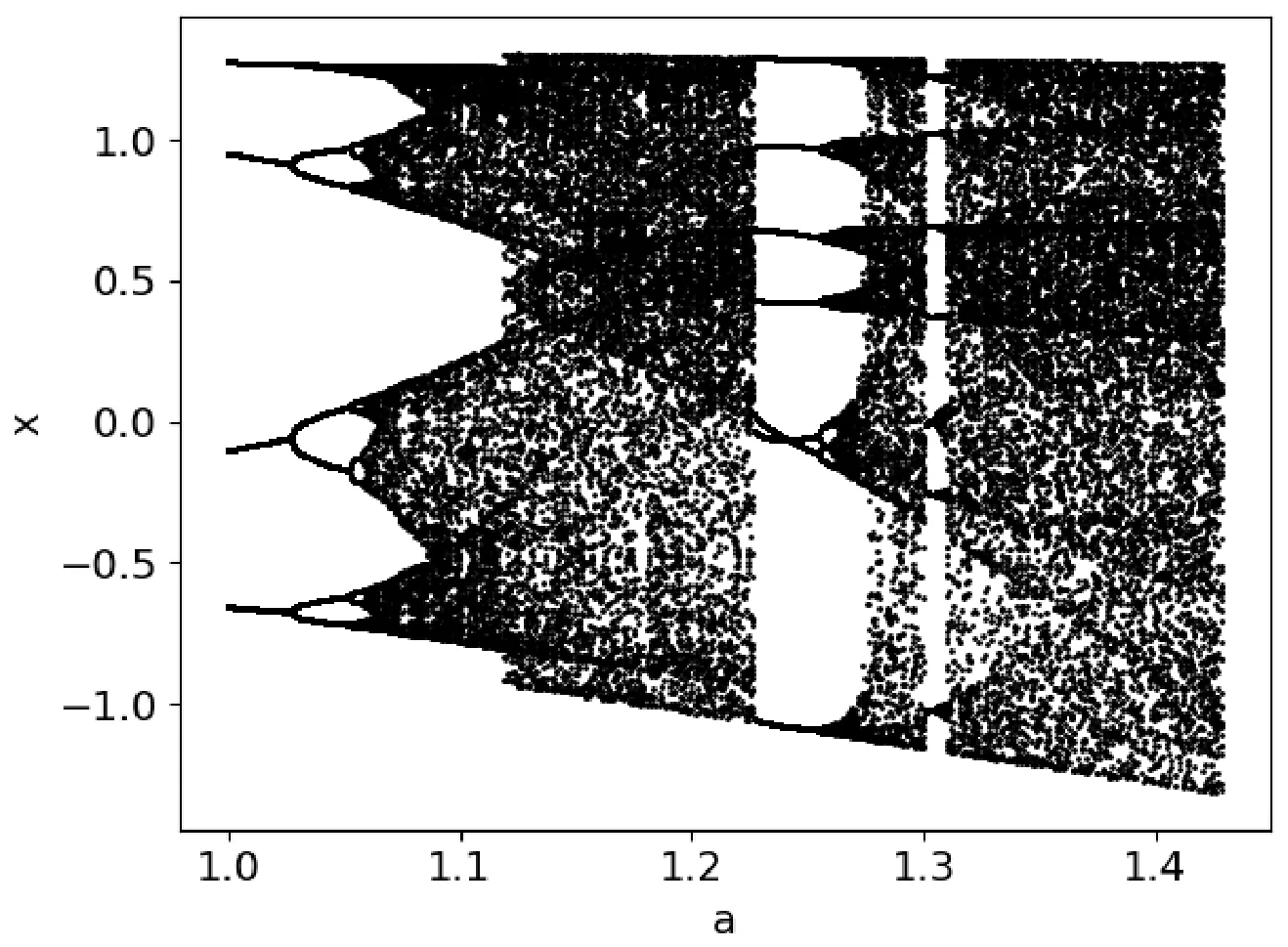
Bifurcation diagram of the Hénon map with initial conditions $x_{0}=y_{0}=0.2$, 200 iterations and b=0.3 for each *a*.

**Figure 4:**
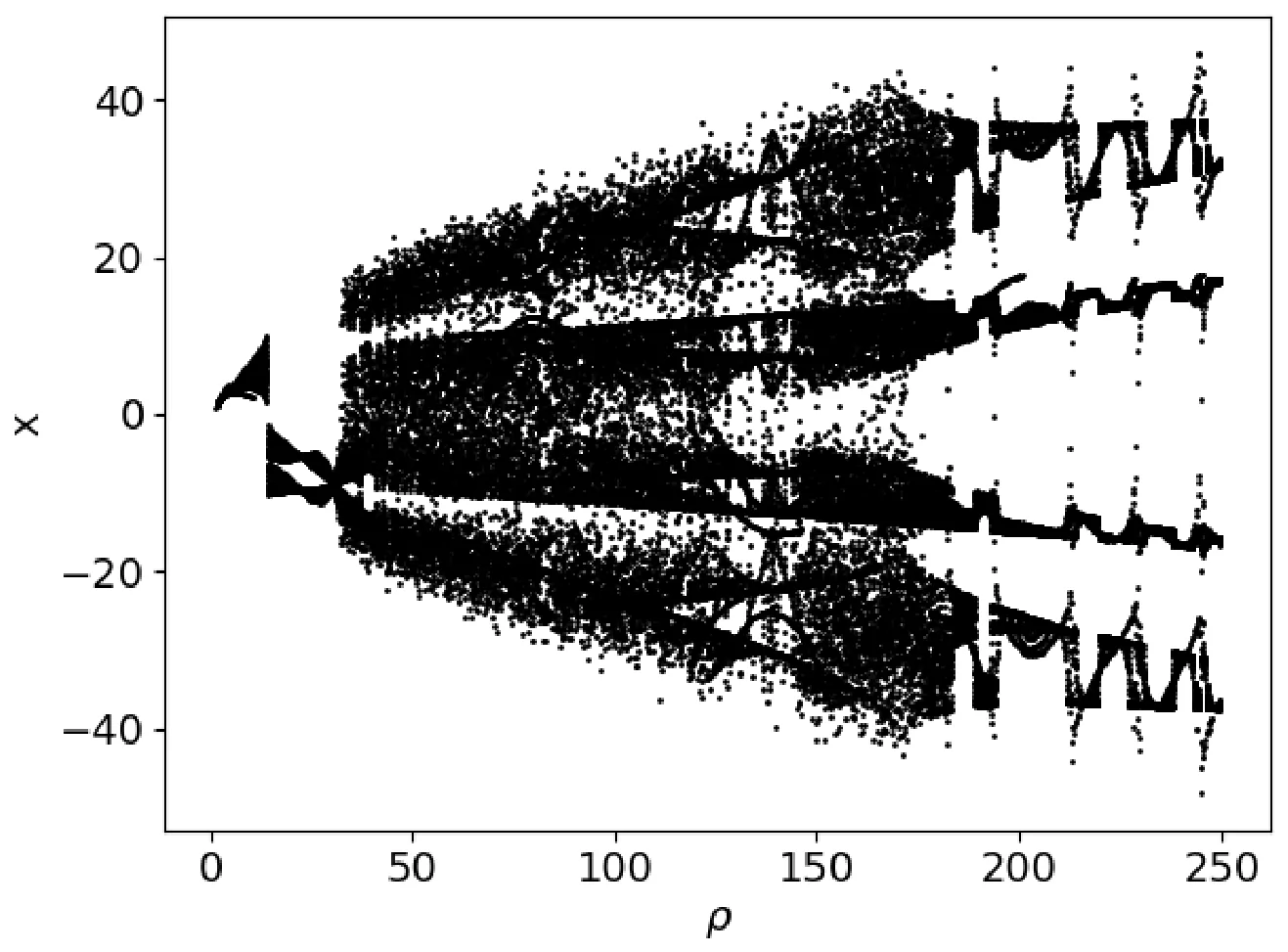
Bifurcation diagram of the Lorenz system with the initial condition $x_{0}=0$, $y_{0}=1$ and $z_{0}=1.05$, 10,000 iterations, β=8/3, σ=10 and Δt=0.001 for each ρ. A Poincaré section at z=ρ−1 was used.

**Figure 5:**
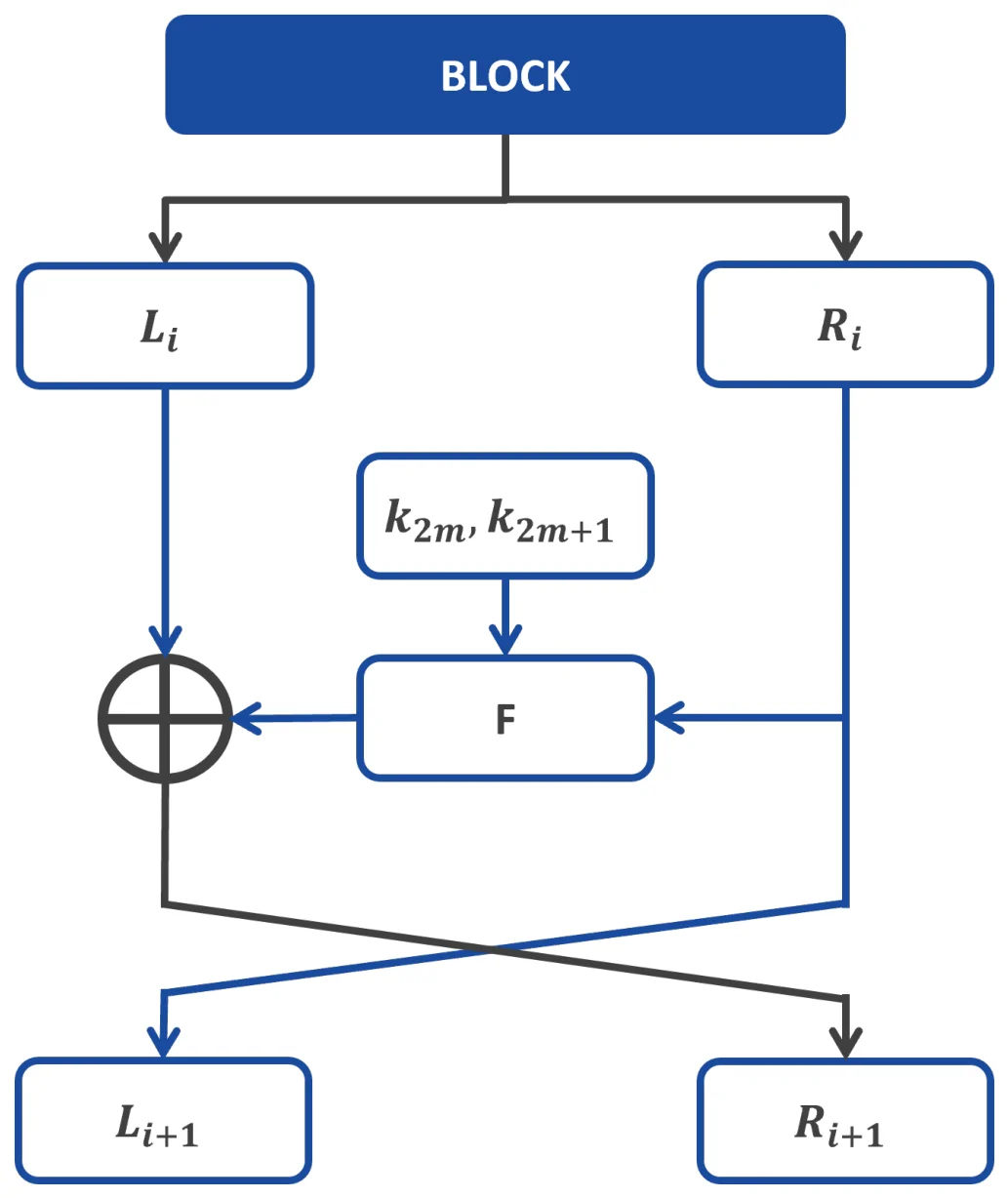
Example of a Feistel network. The input block is first divided into the left-hand block $L_{i}$ and right-hand block $R_{i}$. The two blocks are then processed using XOR and *F* operations to produce $L_{i+1}$ and $R_{i+1}$.

**Figure 6:**
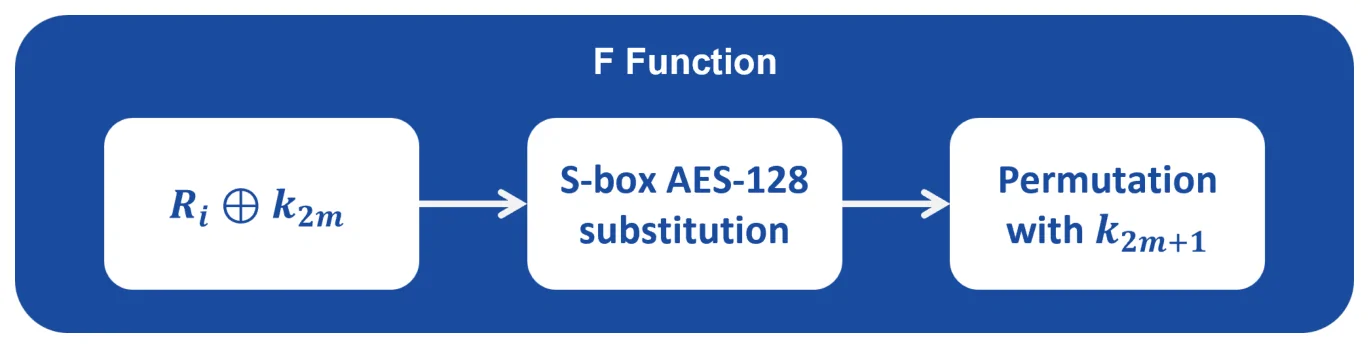
Scheme of cryptography function *F* computation.

**Figure 7:**
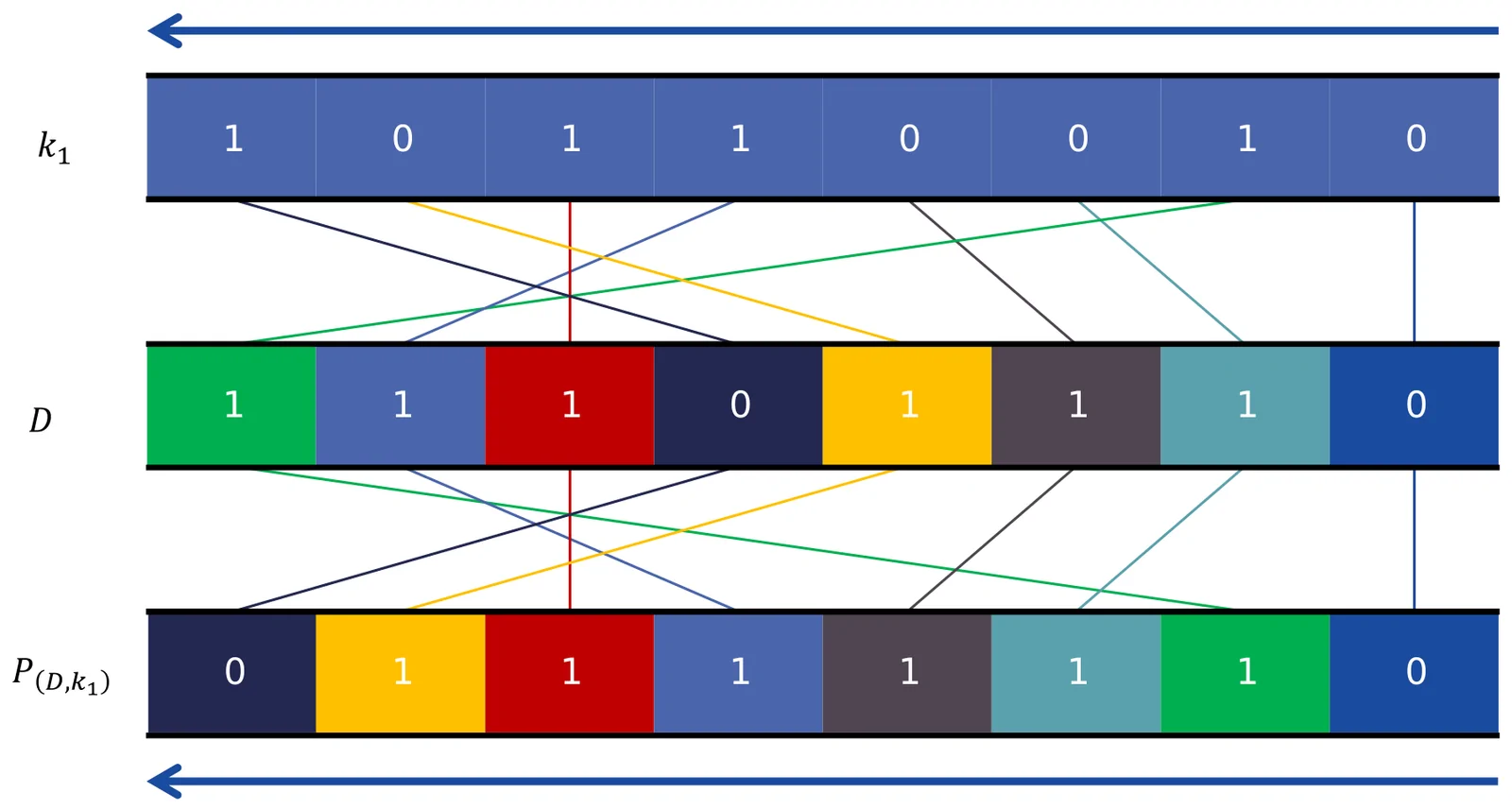
Example of permutation process $P(D,k_{1})$ proposed by [15] considering the key $k_{1}$ and the data *D*.

**Figure 8:**
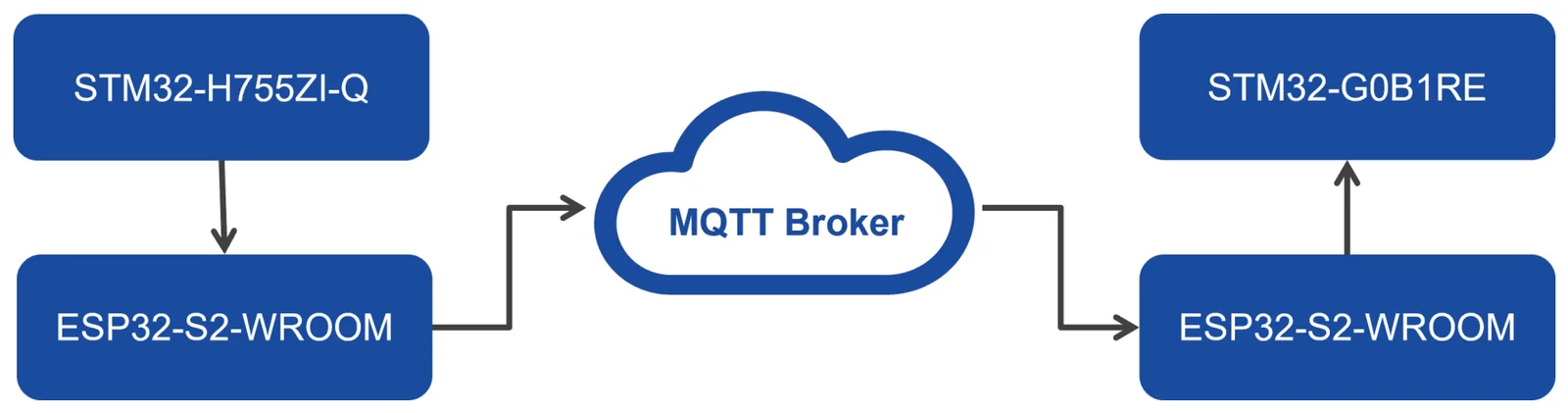
Example of communication tests in order to encrypt and decrypt messages in different devices. In this example, the Cortex-M7 on the left encrypts the message, sends it to the ESP32 via serial communication, and the ESP32 publishes the encrypted message on a topic of an MQTT server. Then the ESP32 on the right subscribes to the MQTT topic of the MQTT, receives the encrypted message, transmits it to the Cortex-M0+ via serial, and then the device performs the decryption of the data.

**Figure 9:**
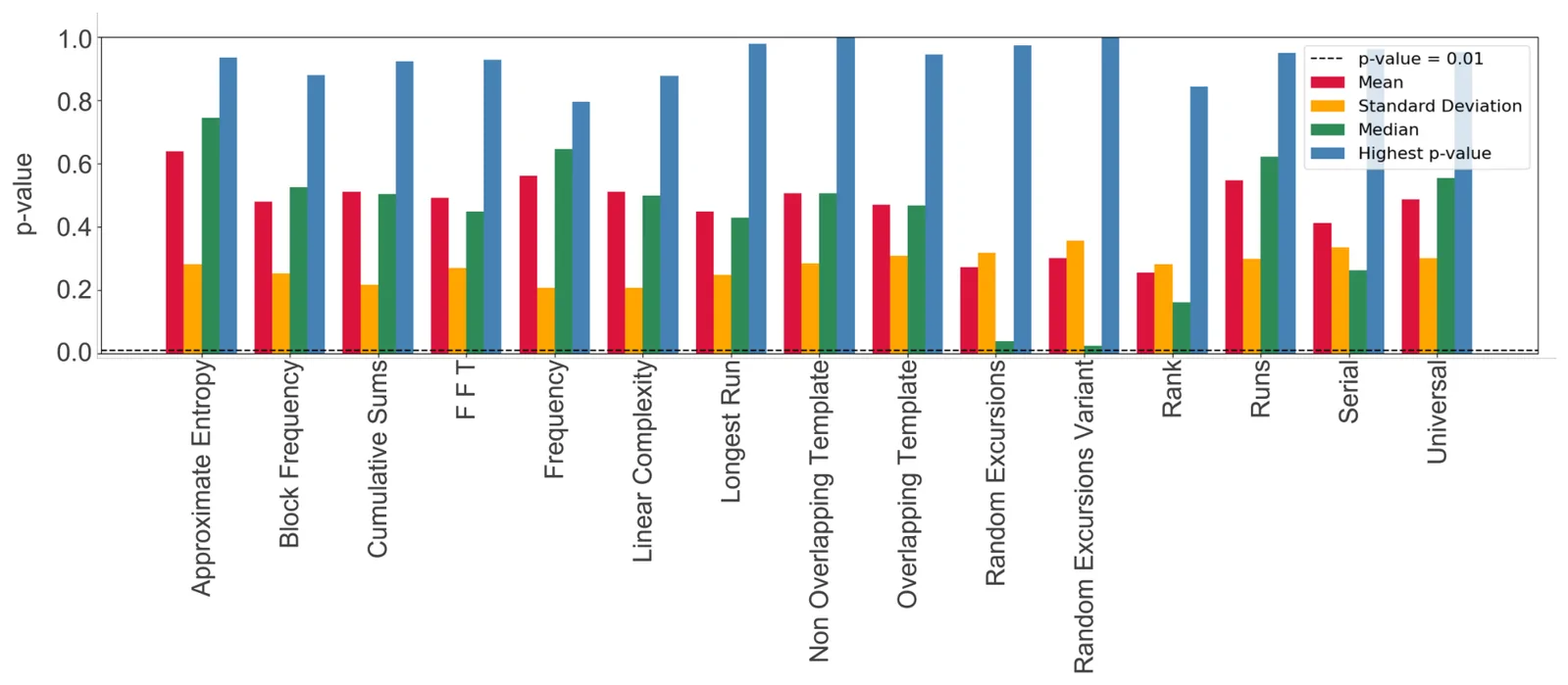
NIST tests considering the logistic map with initial conditions $0.01\leq x_{0}<1$, 3.9≤r<4, and 1000 iterations.

**Figure 10:**
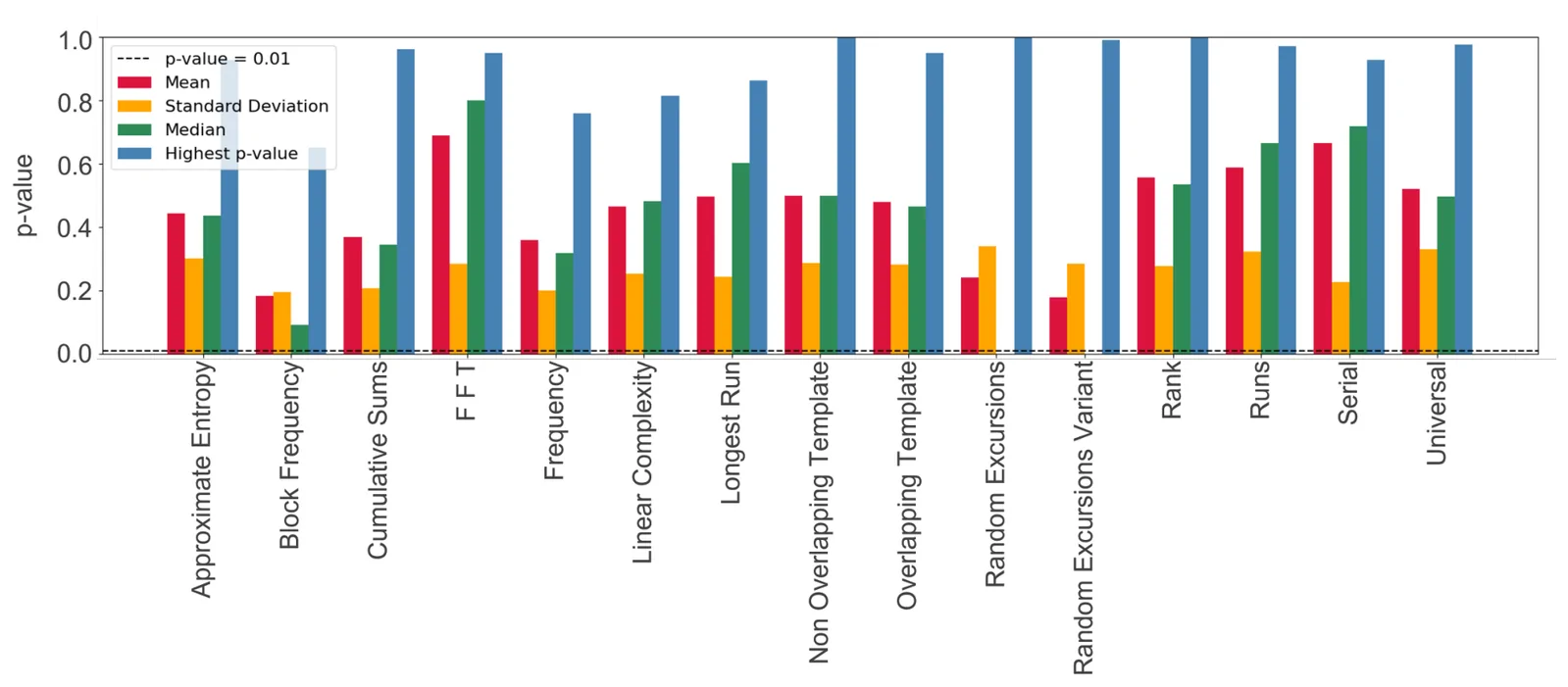
NIST tests considering the Hénon map with the initial condition range $0.02\leq x_{0}<1.02$, $0.02\leq y_{0}<0.2$, parameter range 1.4≤a<1.41 and b=0.3, and 1000 iterations.

**Figure 11:**
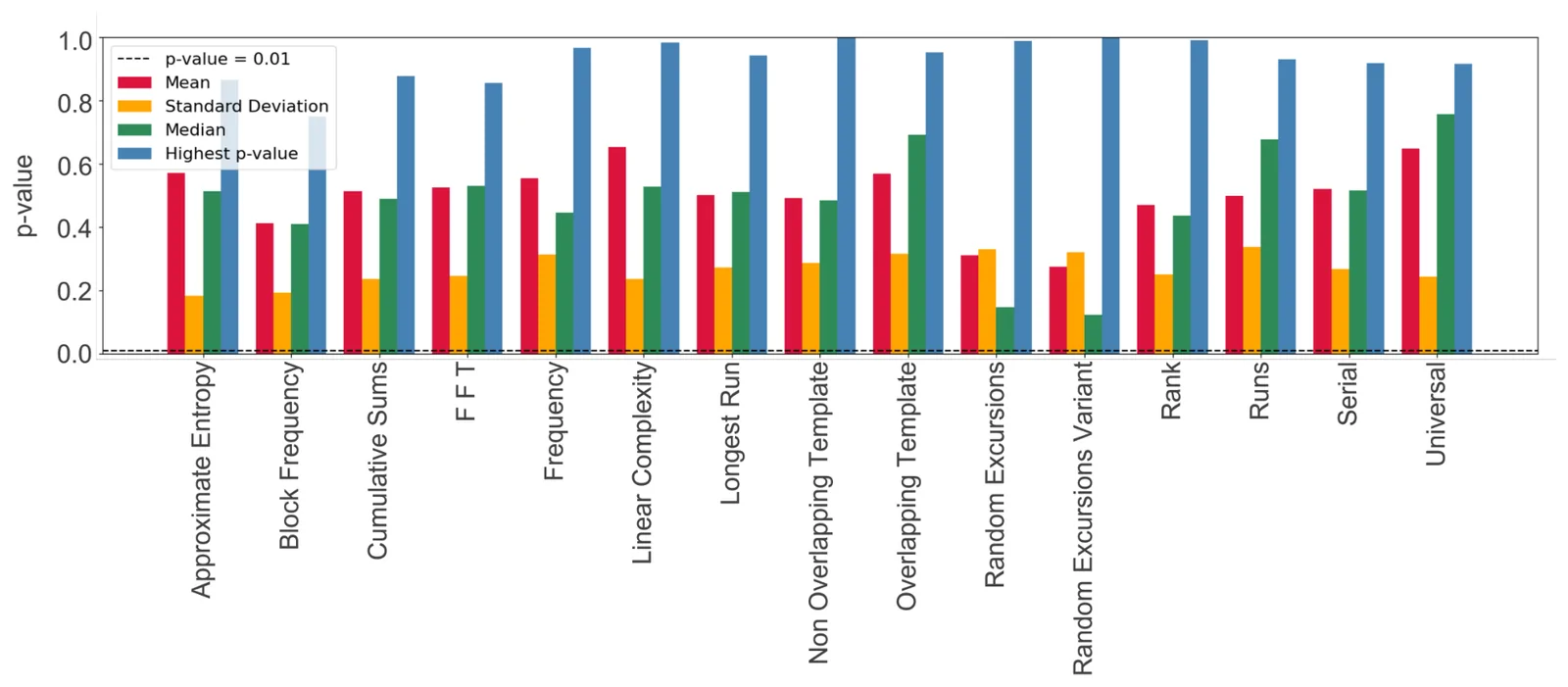
NIST tests considering the Lorenz system with the initial condition range $1\leq x_{0}<1.5$, $1\leq y_{0}<1.3$, $1.05\leq z_{0}<1.25$, 28≤ρ<30, β=10/3, σ=10, 1000 iterations, and Δt=0.01.

**Figure 12:**
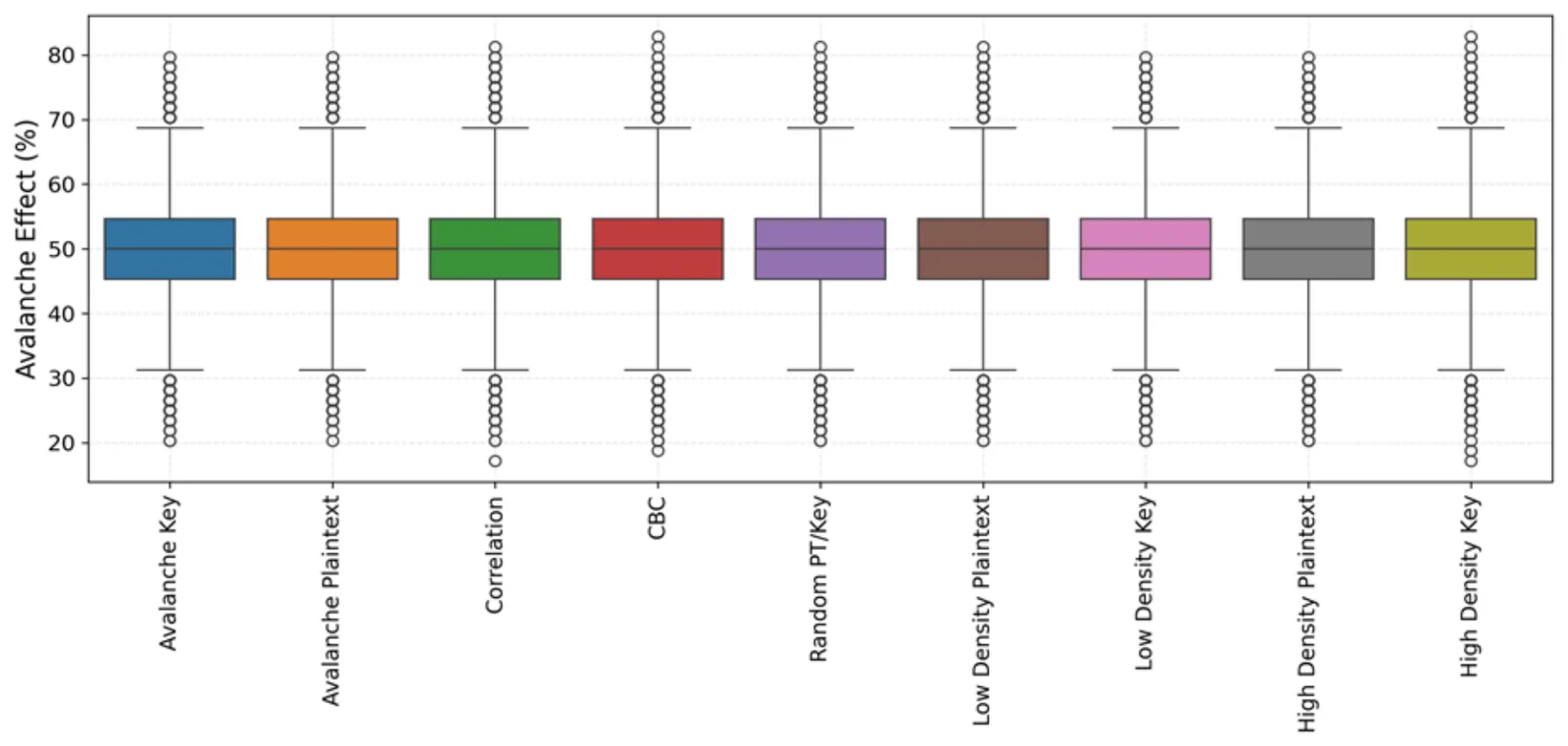
Avalanche effect test results for CHIMA 64-bit.

**Figure 13:**
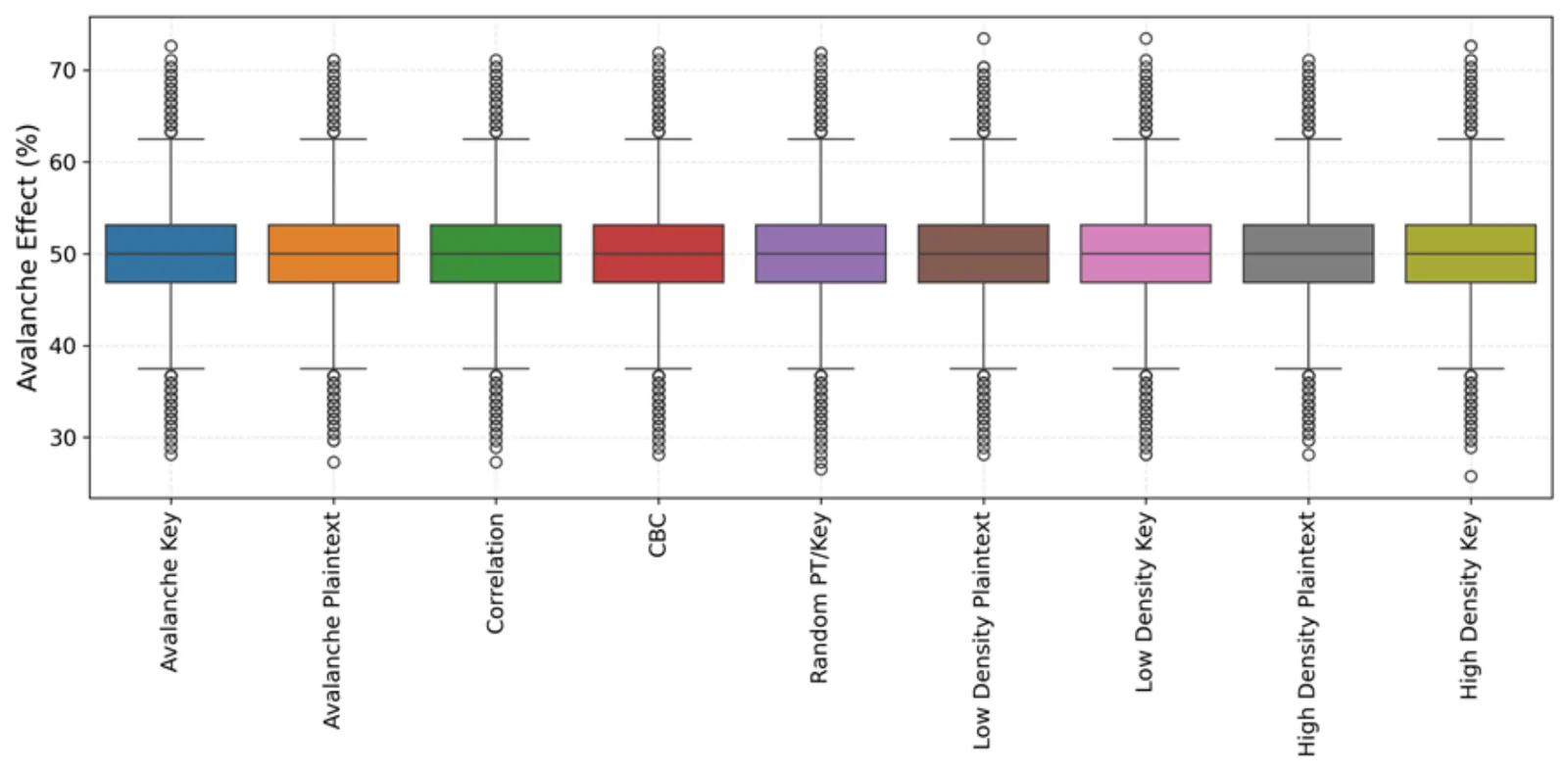
Avalanche effect test results for CHIMA 128-bit.

**Figure 14:**
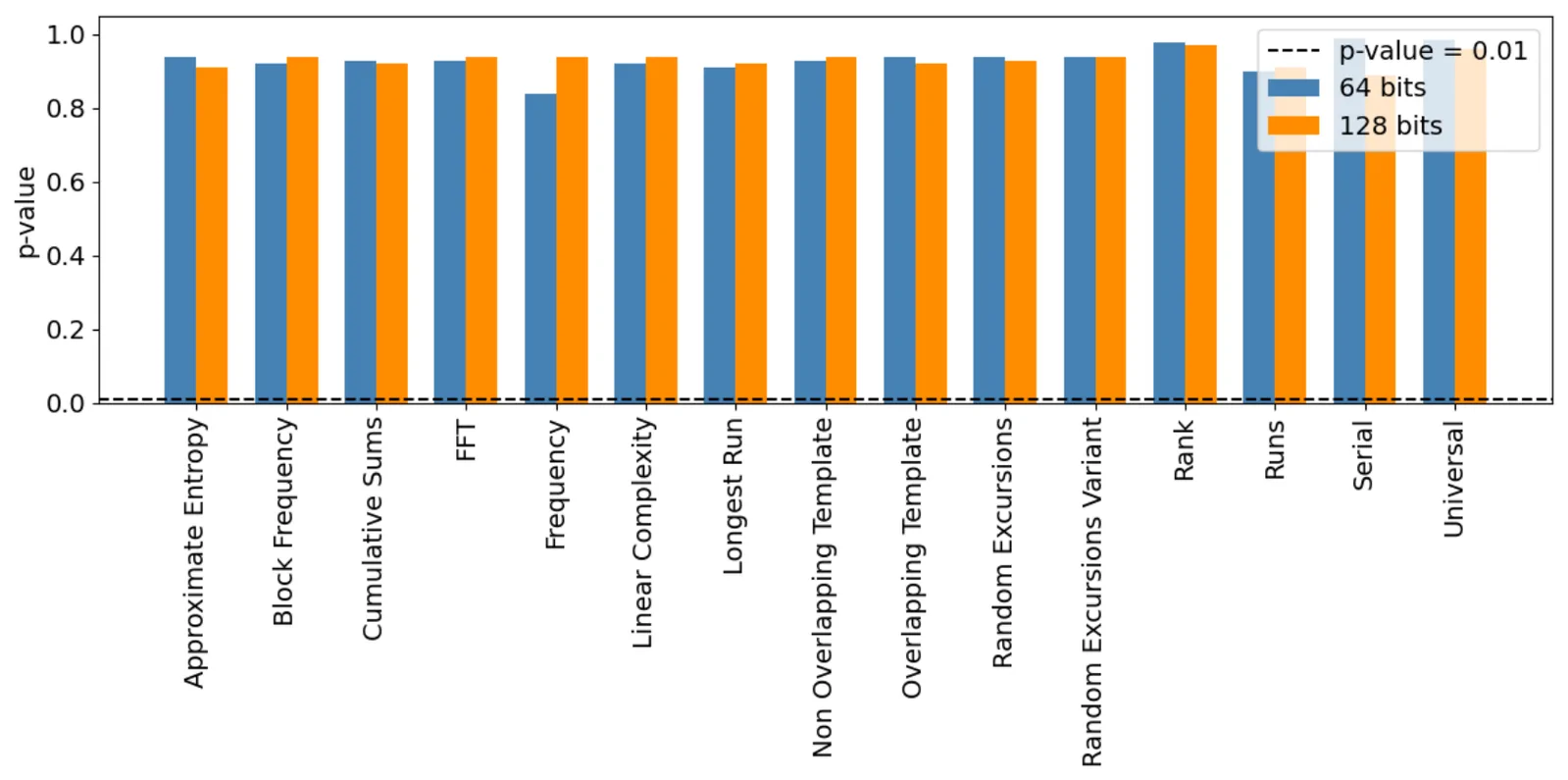
Statistical test results for the CHIMA 64-bit and 128-bit versions. The values presented are the highest values for each test, as it is common to present in the literature.

**Table 1:** Example of usage for the S-box AES-128. MSN stands for Most Significant Nibble, while LSN stands for Least Significant Nibble.

MSN\LSN	00	01	02	03	04	05	06	07	08	09	0A	0B	0C	0D	0E	0F
00	63	7C	77	7B	F2	6B	6F	C5	30	01	67	2B	FE	D7	AB	76
10	CA	82	C9	7D	FA	59	47	F0	AD	D4	A2	AF	9C	A4	72	C0
20	B7	FD	93	26	36	3F	F7	CC	34	A5	E5	F1	71	D8	31	15
30	04	C7	23	C3	18	96	05	9A	07	12	80	E2	EB	27	B2	75
40	09	83	2C	1A	1B	6E	5A	A0	52	3B	D6	B3	29	E3	2F	84
50	53	D1	00	ED	20	FC	B1	5B	6A	CB	BE	39	4A	4C	58	CF
60	D0	EF	AA	FB	43	4D	33	85	45	F9	02	7F	50	3C	9F	A8
70	51	A3	40	8F	92	9D	38	F5	BC	B6	DA	21	10	FF	F3	D2
80	CD	0C	13	EC	5F	97	44	17	C4	A7	7E	3D	64	5D	19	73
90	60	81	4F	DC	22	2A	90	88	46	**EE**	B8	14	DE	5E	0B	DB
A0	E0	32	3A	0A	49	06	24	5C	C2	D3	AC	62	91	95	E4	79
B0	E7	C8	37	6D	8D	D5	4E	A9	6C	56	F4	EA	65	7A	AE	08
C0	BA	78	25	2E	1C	A6	B4	C6	E8	DD	74	1F	4B	BD	8B	8A
D0	70	3E	B5	66	48	03	F6	0E	61	35	57	B9	86	C1	1D	9E
E0	E1	F8	98	11	69	D9	8E	94	9B	1E	87	E9	CE	55	28	DF
F0	8C	A1	89	0D	BF	E6	42	68	41	99	2D	0F	B0	54	BB	16

**Table 2:** Time for the computation of 1000 iterations of the three chaotic systems considering Cortex-M0+, Cortex-M33, and Cortex-M7 devices. The results are given in μs (mean ± measurement uncertainty).

Chaotic System	Cortex-M0+	Cortex-M33	Cortex-M7
Logistic map	8635.80 ± 5.78	1301.69 ± 11.24	835.84 ± 3.08
Hénon map	16,666.20 ± 55.69	3278.94 ± 17.37	2268.46 ± 2.74
Lorenz system	242,082.20 ± 62.81	36,573.41 ± 5.80	26,430.38 ± 23.22

**Table 3:** Execution time of the complete 128-bit master key-generation routine (logistic map iterations and post-processing key derivation) integrated within the final firmware stack.

Device	Time (μs)
Cortex-M0+	8472.00
Cortex-M33	1376.73
Cortex-M7	1020.62

**Table 4:** Energy consumption of the complete 128-bit master key-generation routine (logistic map iterations and post-processing key derivation) integrated within the final firmware stack.

Device	Energy (μJ)
Cortex-M0+	19.752
Cortex-M33	9.370
Cortex-M7	11.147

**Table 5:** Results of functional tests for CHIMA 64-bit and 128-bit versions.

Test	Result
Known Answer Test	Approved
Monte Carlo Test	Approved
Multi-Block Message	Approved
Operation Mode	Approved

**Table 6:** Time consumption of the 64-bit algorithms for encryption (Enc.) and decryption (Dec.) on three different devices. The time is given in μs.

Algorithm	Cortex-M0+		Cortex-M33		Cortex-M7	
	Enc.	Dec.	Enc.	Dec.	Enc.	Dec.
CHIMA 64 (this work)	1209.00	1208.00	303.96	303.07	246.60	245.48
PRESENT 64	3333.00	3213.00	840.04	853.79	573.84	596.30

**Table 7:** Time consumption of the 128-bit algorithms for encryption (Enc.) and decryption (Dec.) on three different devices. The time is given in μs.

Algorithm	Cortex-M0+		Cortex-M33		Cortex-M7	
	Enc.	Dec.	Enc.	Dec.	Enc.	Dec.
LEA 128	296.00	307.00	80.68	84.21	82.17	85.79
ASCON 128	1334.00	1338.00	236.25	238.26	269.49	270.36
CHIMA 128 (this work)	2493.00	2492.00	506.97	506.31	380.53	381.16
CLEFIA 128	2875.00	2876.00	715.68	715.16	719.84	720.15
AES 128	998.00	5527.00	289.44	1392.15	301.92	1572.41

**Table 8:** Energy consumption of the 64-bit algorithms on the three devices.

Algorithm	Energy (μJ)					
	Cortex-M0+		Cortex-M33		Cortex-M7	
	Enc.	Dec.	Enc.	Dec.	Enc.	Dec.
CHIMA 64 (this work)	3.182	3.155	2.588	2.543	3.214	3.159
PRESENT 64	8.121	8.290	6.895	6.561	7.341	7.526

**Table 9:** Energy consumption of the 128-bit algorithms on the three devices.

Algorithm	Energy (μJ)					
	Cortex-M0+		Cortex-M33		Cortex-M7	
	Enc.	Dec.	Enc.	Dec.	Enc.	Dec.
LEA 128	0.847	0.854	0.910	0.883	1.265	1.287
ASCON 128	3.540	3.547	2.216	2.293	3.457	3.468
CHIMA 128 (this work)	4.999	4.987	4.071	4.101	4.931	4.990
CLEFIA 128	6.830	6.862	5.611	5.571	7.845	7.808
AES 128	2.571	14.354	2.394	10.365	3.630	17.625

**Table 10:** Memory usage of each algorithm on each device.

Algorithm	Device	RAM (KB)	FLASH (KB)
AES 128	Cortex-M0+	2.23	23.29
	Cortex-M33	2.32	34.82
	Cortex-M7	2.38	36.63
ASCON 128	Cortex-M0+	2.30	23.85
	Cortex-M33	2.45	38.21
	Cortex-M7	2.45	36.91
LEA 128	Cortex-M0+	2.91	20.71
	Cortex-M33	3.07	35.99
	Cortex-M7	3.08	34.70
CLEFIA 128	Cortex-M0+	2.87	22.34
	Cortex-M33	3.02	37.16
	Cortex-M7	3.02	35.73
PRESENT 64	Cortex-M0+	2.18	19.88
	Cortex-M33	2.62	36.20
	Cortex-M7	2.62	34.90
CHIMA 64 and 128 *	Cortex-M0+	2.49	27.81
	Cortex-M33	2.62	39.34
	Cortex-M7	2.62	38.04

* Both the CHIMA-64 and CHIMA-128 variants share the same core functional routines within a unified firmware build; hence, memory consumption is reported for the integrated binary.

**Table 11:** Time consumption of the integrity check on the three devices.

Device	Time (μs)
Cortex-M0+	8302.00
Cortex-M33	2465.80
Cortex-M7	2317.87

**Table 12:** Energy consumption of the integrity check on the three devices.

Device	Energy (μJ)
Cortex-M0+	19.97
Cortex-M33	18.48
Cortex-M7	28.65

## Data Availability

The data are available upon reasonable request.
